# Supplementary material for: SERTM2: a neuroactive player in the world of micropeptides
Source: EMBO Rep. 2025 Mar 19;26(8):2044–76. doi: 10.1038/s44319-025-00404-w (PMC12019361; doi:10.1038/s44319-025-00404-w)
Supplement: Supplementary file 1 — Appendix [file 44319_2025_404_MOESM1_ESM.pdf]

APPENDIX

SERTM2: a neuroactive player in the world of micropeptides

Lisi M.[1,2], Santini T. [1,2], D'Andrea T. [3], Salvatori B.[2], Setti A.[1], Paiardini A.[4], Nutarelli S.[1], Nicoletti C.[5], Pellegrini F.[1], Fucile S. [3,6], Bozzoni I.[1,2,7,9*], Martone J.[8,9,*]

[1]Department of Biology and Biotechnologies "Charles Darwin", Sapienza University of Rome, Rome, Italy

[2]Center for Life Nano-& Neuro-Science, Fondazione Istituto Italiano di Tecnologia, Rome, Italy;

[3] IRCCS Neuromed, Pozzilli, Italy

[4] Department of Biochemical Sciences, Sapienza University of Rome, Rome, Italy.

[5] DAHFMO-Unit of Histology and Medical Embryology, Laboratory affiliated to Istituto Pasteur Italia-Fondazione Cenci Bolognetti, Sapienza University of Rome, Rome, Italy

[6] Department of Physiology and Pharmacology "V. Erspamer", Sapienza University of Rome, Rome, Italy;

[7]Center for Human Technologies, Istituto Italiano di Tecnologia, Genoa, Italy;

[8]Institute of Molecular Biology and Pathology, National Research Council, Sapienza University of Rome, Rome, Italy.

[9]Lead contact

*Correspondence: irene.bozzoni@uniroma1.it, julie.martone@cnr.it

TABLE OF CONTENT:

Appendix Figure S1 (pag. 2).

Appendix Figure S2 (pag. 3).

Appendix Table S1. Translational initiation site (TIS) prediction (pag. 4).

Appendix Table S2. Ion channels co-expressed with lncMN3 (pag. 5).

Appendix Table S3. Sertm2-KO mice were born in expected Mendelian ratios (pag. 6).

A

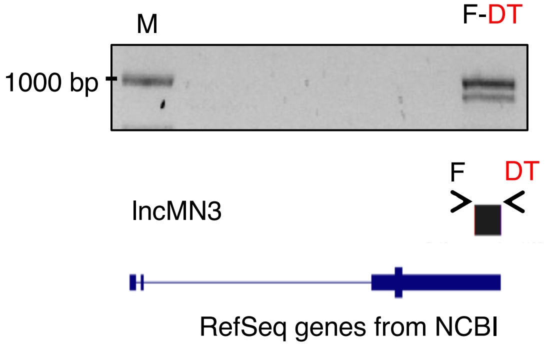

B

Mouse- CPAT prediciton

Sequence Name	RNA Size	ORF Size	Ficket Score	Hexamer Score	Coding Probability	Coding Label
ENSMUST00000135687.2	4707	270	1.1438	0.11899456778	0.25272677702221	no

C

Mouse- CPC prediction

ID	Label	Coding probability	Peptide length(aa)	Fickett score	Isoelectric point	ORF integrity
ENSMUST00000135687.2	noncoding	0.132395	90	0.29676	5.80780029297	complete

D

Human- CPAT prediction

Sequence Name	RNA Size	ORF Size	Ficket Score	Hexamer Score	Coding Probability	Coding Label
ENST00000569275.2	4612	273	1.1003	0.225615642478	0.38257489395799	yes

Appendix Figure S1.

A) Amplification product obtained using 3’RACE kit. Lower panel: UCSC Genome Browser view (GRCm38/mm10 Assembly) of a portion of the murine lncMN3 locus showing the alignment of the sequenced product of the 3’ RACE (3’ rapid amplification of cDNA ends).

B) Coding potential analyses of murine lncMN3 sequence performed with Coding Potential Assessment tool (CPAT)

C) Coding potential analyses of murine lncMN3 sequence performed with Coding Potential Calculator tool (CPC).

D) Coding potential analyses of human SERTM2 sequence performed with CPAT.

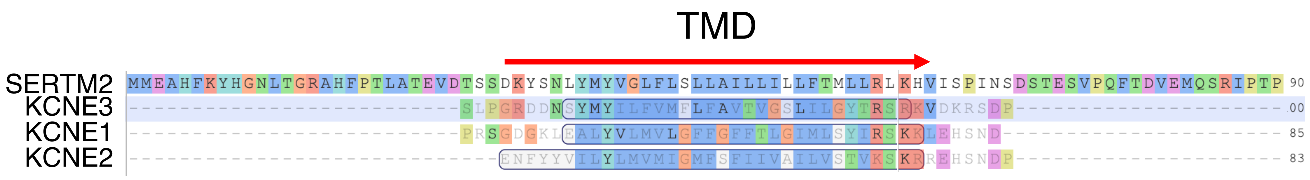

Appendix Figure S2.

Sequence alignment betweenn the TM domain of SERTM2 and representative members of the KCNE family of potassium channel modulators whose 3D structures are known. Residues are colored according to physico-chemical properties.

Predict TIS (https://www.tispredictor.com/)	
Human	Mouse
>ENST00000569275.2 SERTM2-202 cdna:protein_coding	>ENSMUST00000135687.2 Sertm2-201 cdna:protein_coding
Kozak Similarity Scores:	Kozak Similarity Scores:
Position 1014: 0.58	Position 241: 0.6
Position 1074: 0.79	Position 1058: 0.58
Position 1096: 0.78	Position 1115: 0.65
Position 1236: 0.64	Position 1140: 0.74
Position 1255: 0.76	Position 1280: 0.64
Position 1317: 0.64	Position 1299: 0.76
Position 1351: 0.74	Position 1756: 0.69
Position 1391: 0.55	Position 1825: 0.69
Position 1810: 0.58	Position 1901: 0.66
Position 2167: 0.73	Position 1999: 0.65
Position 2223: 0.64	Position 2467: 0.61
Position 2377: 0.61	Position 2498: 0.68
Position 2408: 0.7	Position 2579: 0.66
Position 2480: 0.59	Position 2694: 0.66
Position 2543: 0.63	Position 2780: 0.75
Position 2711: 0.74	Position 2830: 0.72
Position 2763: 0.62	Position 2930: 0.78
Position 2985: 0.71	Position 3089: 0.55
Position 3166: 0.83	Position 3237: 0.58
Position 3303: 0.55	Position 3608: 0.55
Position 3500: 0.62	Position 3639: 0.81
Position 3537: 0.67	Position 3845: 0.66
Position 3673: 0.66	Position 4272: 0.68
Position 3775: 0.46	Position 4484: 0.65
Position 4029: 0.62	Position 4563: 0.65
Position 4168: 0.75	Position 4592: 0.74
Position 4182: 0.68	
Position 4258: 0.7	
Position 4453: 0.66	
Position 4535: 0.69	
Position 4594: 0.65	

Appendix TABLE S1. Translational initiation site (TIS) prediction. Kozak similarity scores for predicted TIS for mouse and human SERTM2 transcripts. The positions corresponding to the expected ATG start codon of human and mouse SERTM2 are highlighted in orange boxes.

Gene stable ID	MGI description	Level _0	Genes
ENSMUSG00000075316	sodium channel, voltage-gated, type IX, alpha	7	Scn9a
ENSMUSG00000049281	sodium channel, voltage-gated, type III, beta	76	Scn3b
ENSMUSG00000007041	chloride intracellular channel 1	184	Clic1
ENSMUSG00000057182	sodium channel, voltage-gated, type III, alpha	218	Scn3a
ENSMUSG00000019146	calcium channel, voltage-dependent, gamma subunit 2	313	Cacng2
ENSMUSG00000033854	potassium channel, subfamily K, member 10	339	Kcnk10
ENSMUSG00000036667	TRPM8 channel-associated factor 1	341	Tcaf1
ENSMUSG00000004110	calcium channel, voltage-dependent, R type, alpha 1E subunit	378	Cacna1e
ENSMUSG00000004113	calcium channel, voltage-dependent, N type, alpha 1B subunit	390	Cacna1b
ENSMUSG00000020723	calcium channel, voltage-dependent, gamma subunit 4	393	Cacng4
ENSMUSG00000018507	transient receptor potential cation channel, subfamily V, member 2	394	Trpv2
ENSMUSG00000063142	potassium large conductance calcium-activated channel, subfamily M, alpha member 1	402	Kcnma1
ENSMUSG00000003352	calcium channel, voltage-dependent, beta 3 subunit	413	Cacnb3
ENSMUSG00000016346	potassium voltage-gated channel, subfamily Q, member 2	453	Kcnq2
ENSMUSG00000033007	acid-sensing (proton-gated) ion channel family member 4	465	Asic4
ENSMUSG00000020704	acid-sensing (proton-gated) ion channel 2	474	Asic2
ENSMUSG00000020882	calcium channel, voltage-dependent, beta 1 subunit	599	Cacnb1
ENSMUSG00000053519	Kv channel-interacting protein 1	648	Kcnip1
ENSMUSG00000000605	chloride channel, voltage-sensitive 4	667	Clcn4
ENSMUSG00000035640	calcium channel, voltage-dependent, beta subunit associated regulatory protein	753	Cbarp
ENSMUSG00000098557	potassium channel tetramerisation domain containing 12	789	Kctd12
ENSMUSG00000036760	potassium channel, subfamily K, member 9	817	Kcnk9
ENSMUSG00000031576	potassium channel, subfamily U, member 1	885	Kcnu1
ENSMUSG00000020402	voltage-dependent anion channel 1	900	Vdac1
ENSMUSG00000049265	potassium channel, subfamily K, member 3	918	Kcnk3
ENSMUSG00000054934	potassium large conductance calcium-activated channel, subfamily M, beta member 4	929	Kcnmb4
ENSMUSG00000075318	sodium channel, voltage-gated, type II, alpha	952	Scn2a

Appendix Table S2. Ion channels co-expressed with lncMN3. List of ion channels co-expressed with lncMN3 according to single cell analysis.

	Females	Males	TOT
Observed number	83	78	161
Expected number	80.5	80.5	
Expected ratio	0.5	0.5	
Actual ratio	0.52	0.48	
	χ2= 0,078		

FEMALES from HET (F) x HEMI (M)	WT	HET	HOMO	TOT
Observed number	0	24	15	39
Expected number	0	19.5	19.5	
Expected ratio	0	0.5	0.5	
Actual ratio	0	0.62	0.38	
	χ2= 1,038			
FEMALES from HET (F) x WT (M)	WT	HET	HOMO	TOT
Observed number	26	18	0	44
Expected number	22	22	0	
Expected ratio	0.5	0.5	0	
Actual ratio	0.59	0.41	0	
	χ2= 0,727			
MALES from HET (F) x HEMI (M)	WT	HOMO	TOT	
Observed number	16	19	35	
Expected number	17.5	17.5		
Expected ratio	0.5	0.5		
Actual ratio	0.46	0.54		
	χ2= 0,128			
MALES from HET (F) x WT (M)	WT	HOMO	TOT	
Observed number	22	21	43	
Expected number	21.5	21.5		
Expected ratio	0.5	0.5		
Actual ratio	0.51	0.49		
	χ2= 0,012			

Appendix Table S3. Sertm2-KO mice were born in expected Mendelian ratios.

The observed ratio did not deviate from the expected ratio in a significative way as shown by the χ^2 test.
