# Supplementary material for: SERTM2: a neuroactive player in the world of micropeptides
Source: EMBO Rep. 2025 Mar 19;26(8):2044–76. doi: 10.1038/s44319-025-00404-w (PMC12019361; doi:10.1038/s44319-025-00404-w)
Supplement: Supplementary file 3 — Source data Fig. 1 [file 44319_2025_404_MOESM3_ESM.zip › Figure 1/1C/README.docx]

All images were acquired as 16 bit depth with a resolution XY of 0,108 micron, by using a UPLANSApo 60X oil objective (NA 1.35) and collected with MetaMorph software (Molecular Devices). Stacks of images were get automatically with 0,2 micron between the Z-slices. All Z stacks were merged with maximum intensity projection and combine in multicolour image. Digital enlargements were obtained by manual cropping from original full field images. Post acquisition processing wad performed by FIJI tools and in particular: “unsharp mask” was used to enhance the signal over background, while “color balance” was manually adjusted to set image background.
