# Supplementary figures and images for: SERTM2: a neuroactive player in the world of micropeptides
Source: EMBO Rep. 2025 Mar 19;26(8):2044–76. doi: 10.1038/s44319-025-00404-w (PMC12019361; doi:10.1038/s44319-025-00404-w)

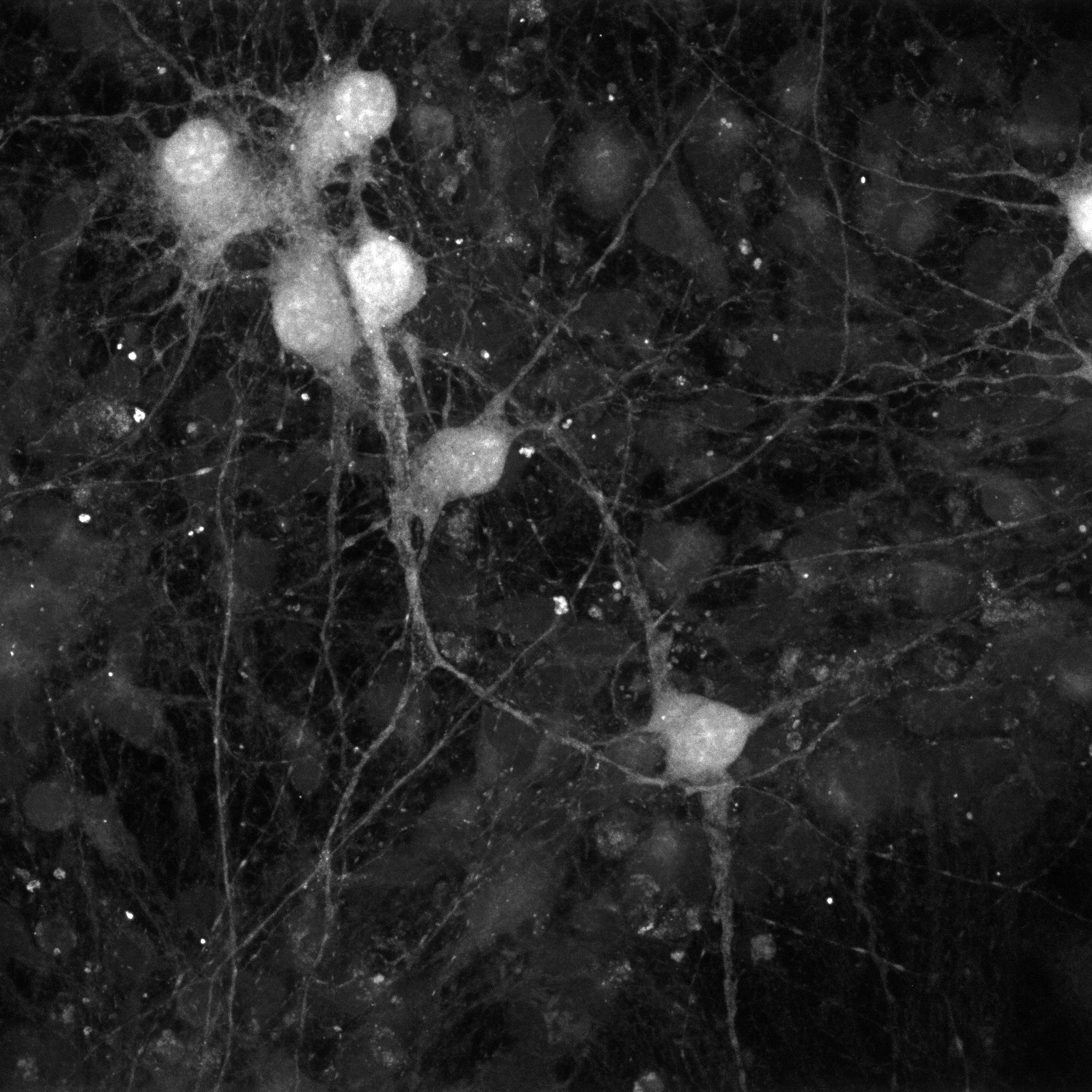

Supplement: Supplementary file 3 — Source data Fig. 1 [file 44319_2025_404_MOESM3_ESM.zip › Figure 1/1C/div 7 source/GFP.tif]

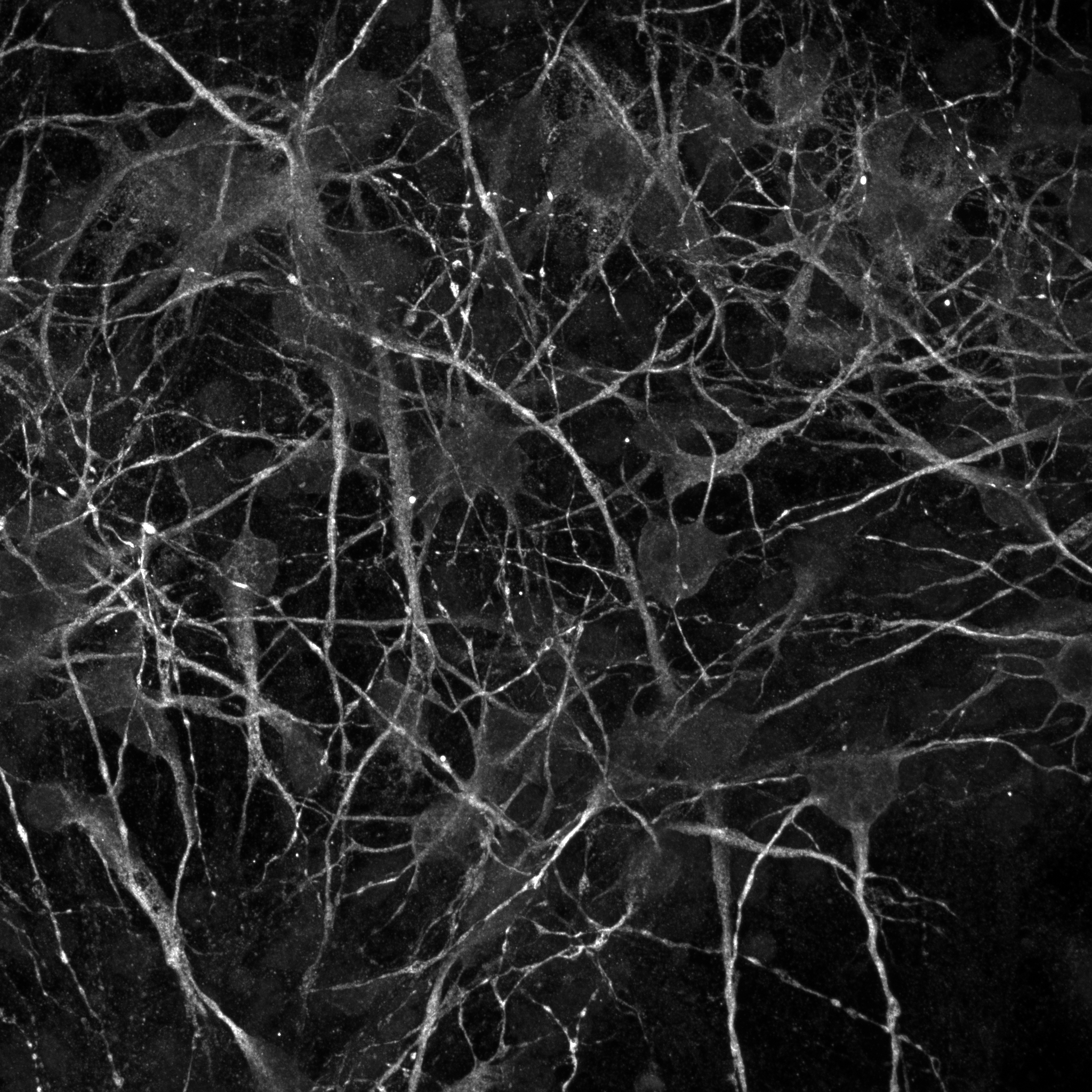

Supplement: Supplementary file 3 — Source data Fig. 1 [file 44319_2025_404_MOESM3_ESM.zip › Figure 1/1C/div 7 source/MAP2.tif]

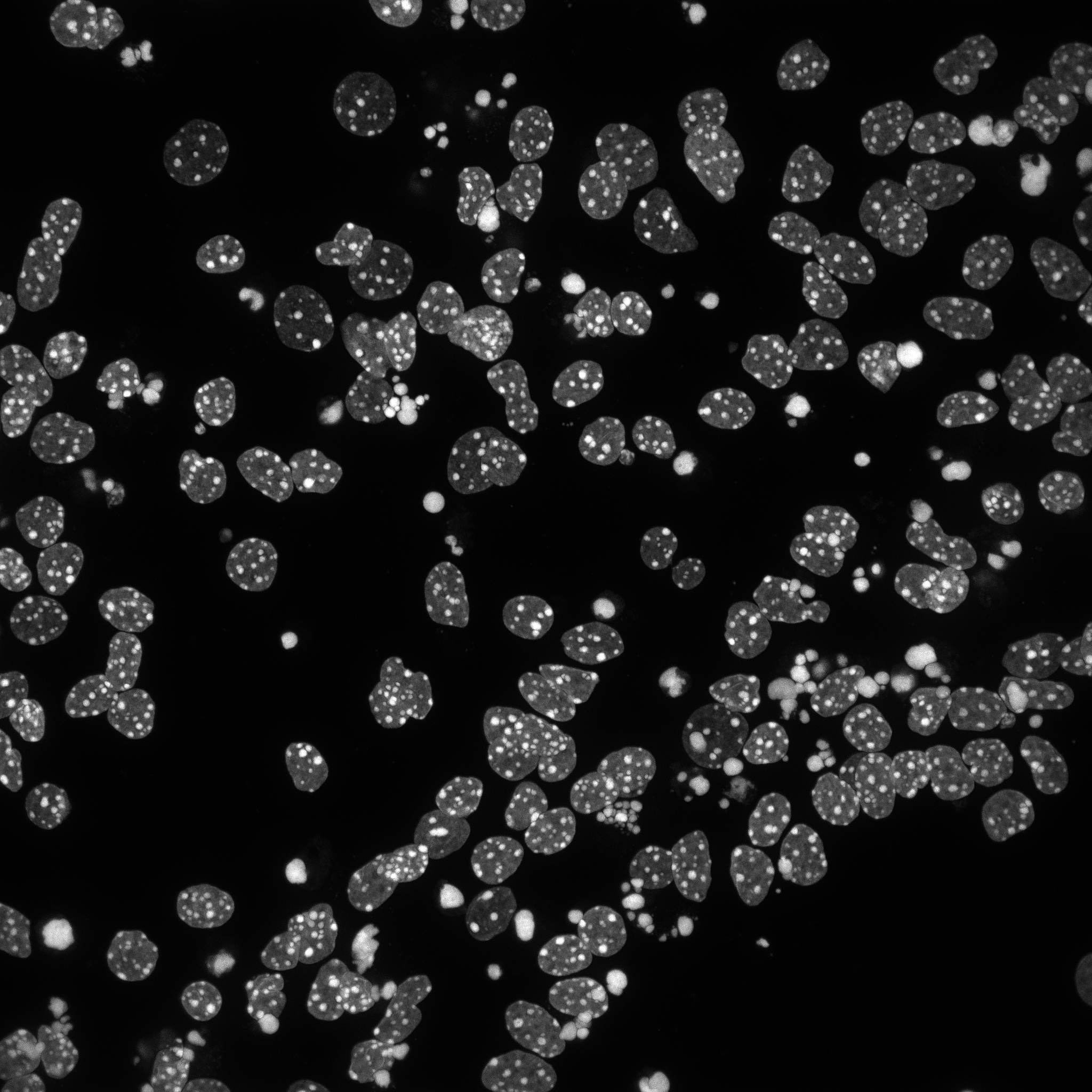

Supplement: Supplementary file 3 — Source data Fig. 1 [file 44319_2025_404_MOESM3_ESM.zip › Figure 1/1C/div 7 source/DAPI.tif]

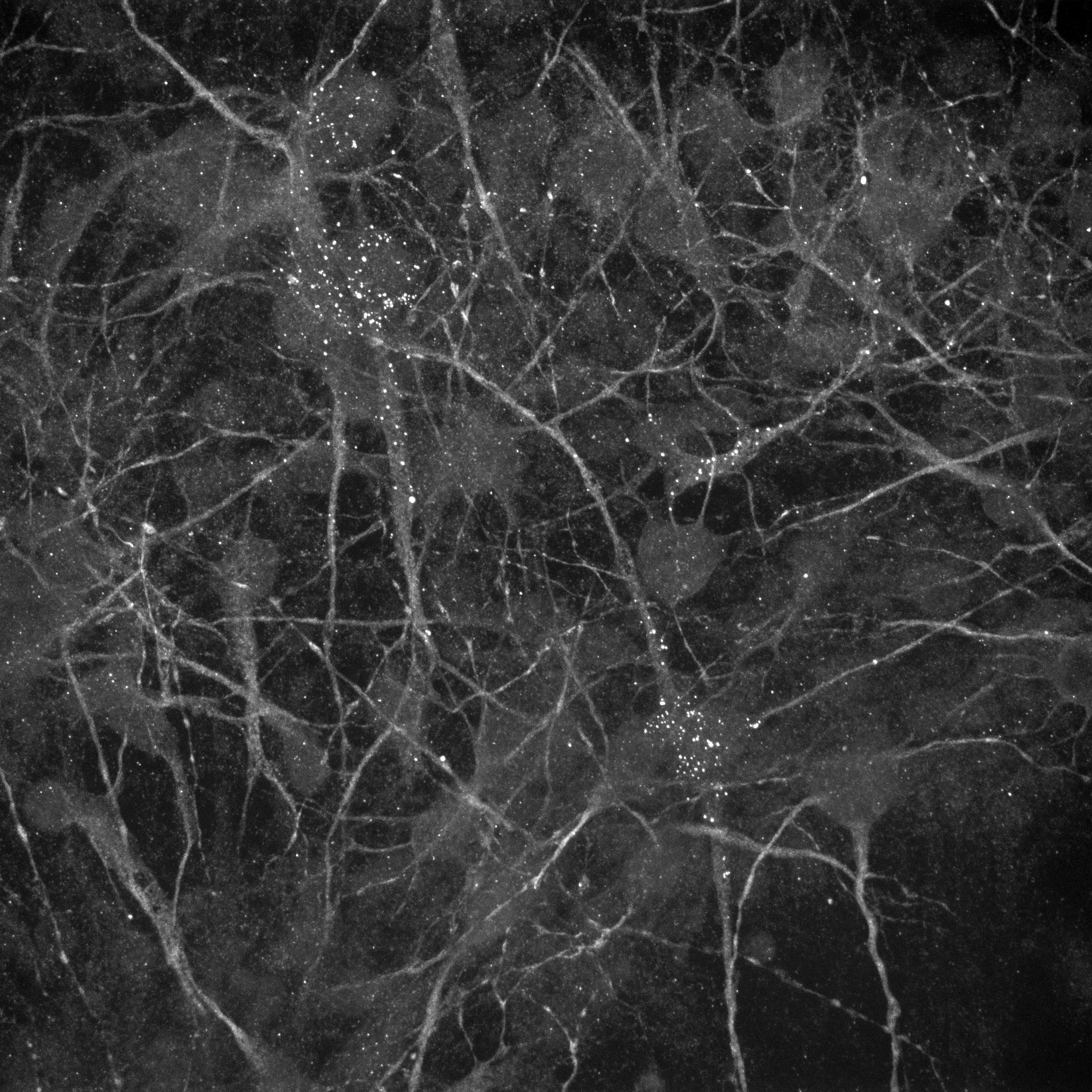

Supplement: Supplementary file 3 — Source data Fig. 1 [file 44319_2025_404_MOESM3_ESM.zip › Figure 1/1C/div 7 source/sertM2.tif]

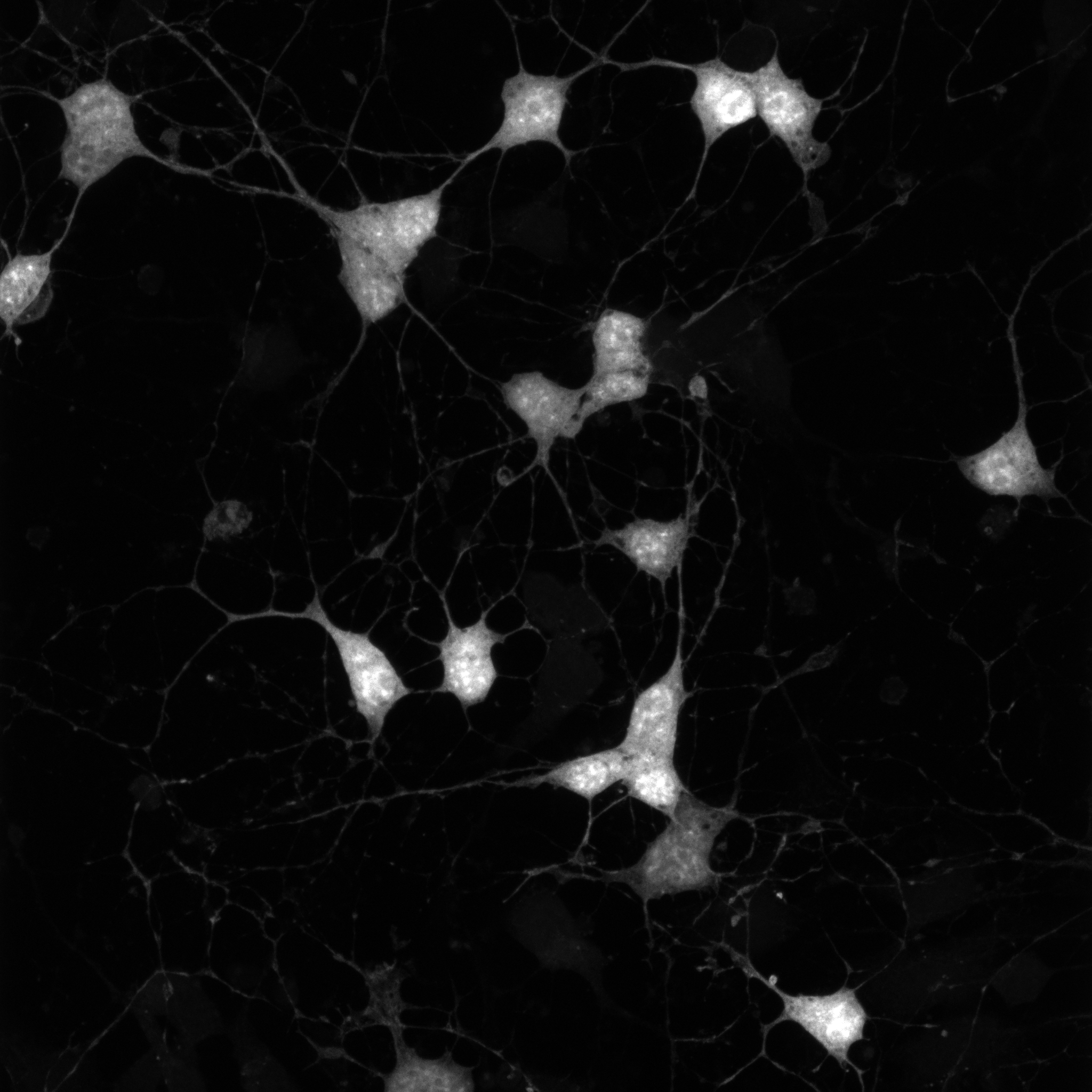

Supplement: Supplementary file 3 — Source data Fig. 1 [file 44319_2025_404_MOESM3_ESM.zip › Figure 1/1C/div1 source/GFP.tif]

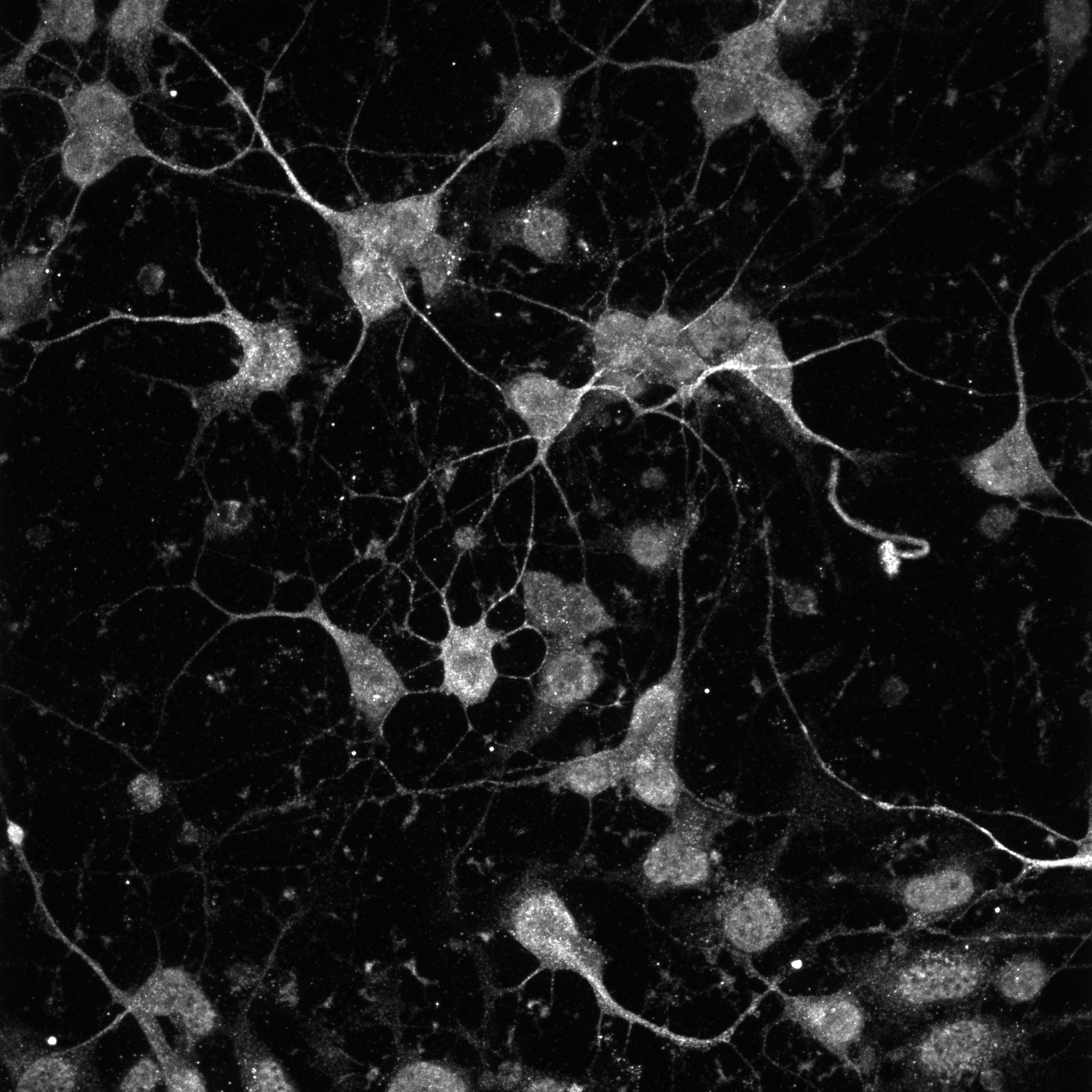

Supplement: Supplementary file 3 — Source data Fig. 1 [file 44319_2025_404_MOESM3_ESM.zip › Figure 1/1C/div1 source/MAP2.tif]

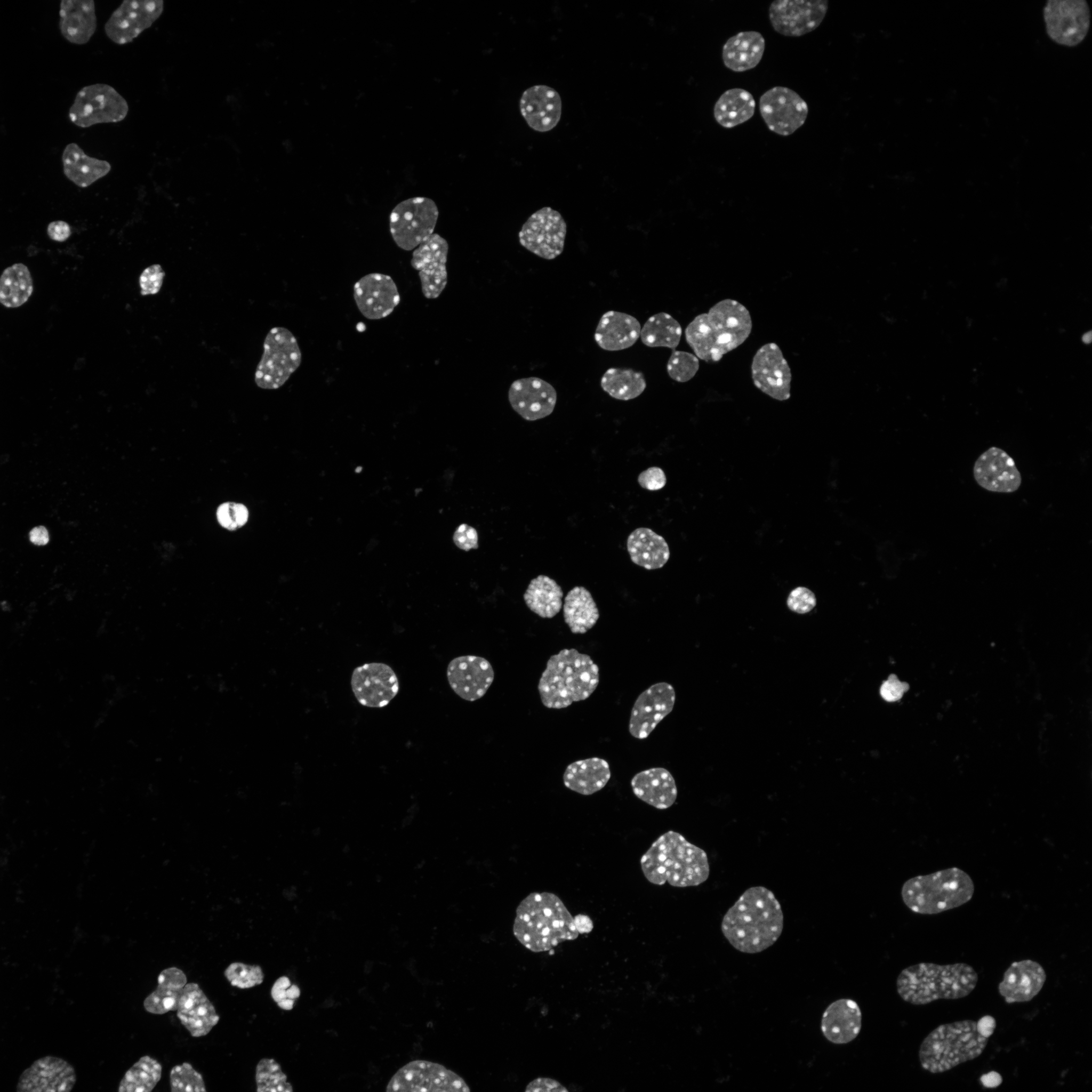

Supplement: Supplementary file 3 — Source data Fig. 1 [file 44319_2025_404_MOESM3_ESM.zip › Figure 1/1C/div1 source/DAPI.tif]

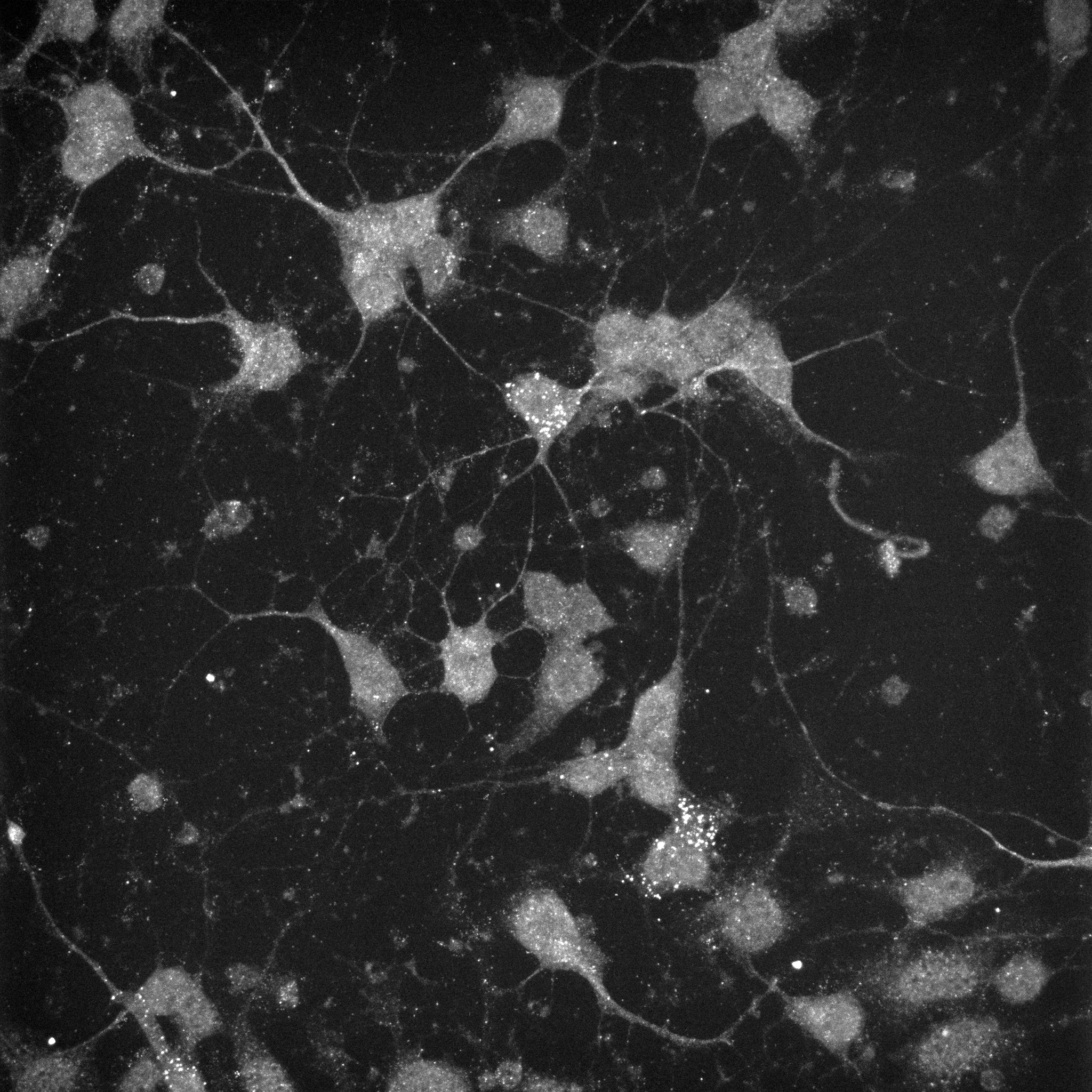

Supplement: Supplementary file 3 — Source data Fig. 1 [file 44319_2025_404_MOESM3_ESM.zip › Figure 1/1C/div1 source/sertM2.tif]

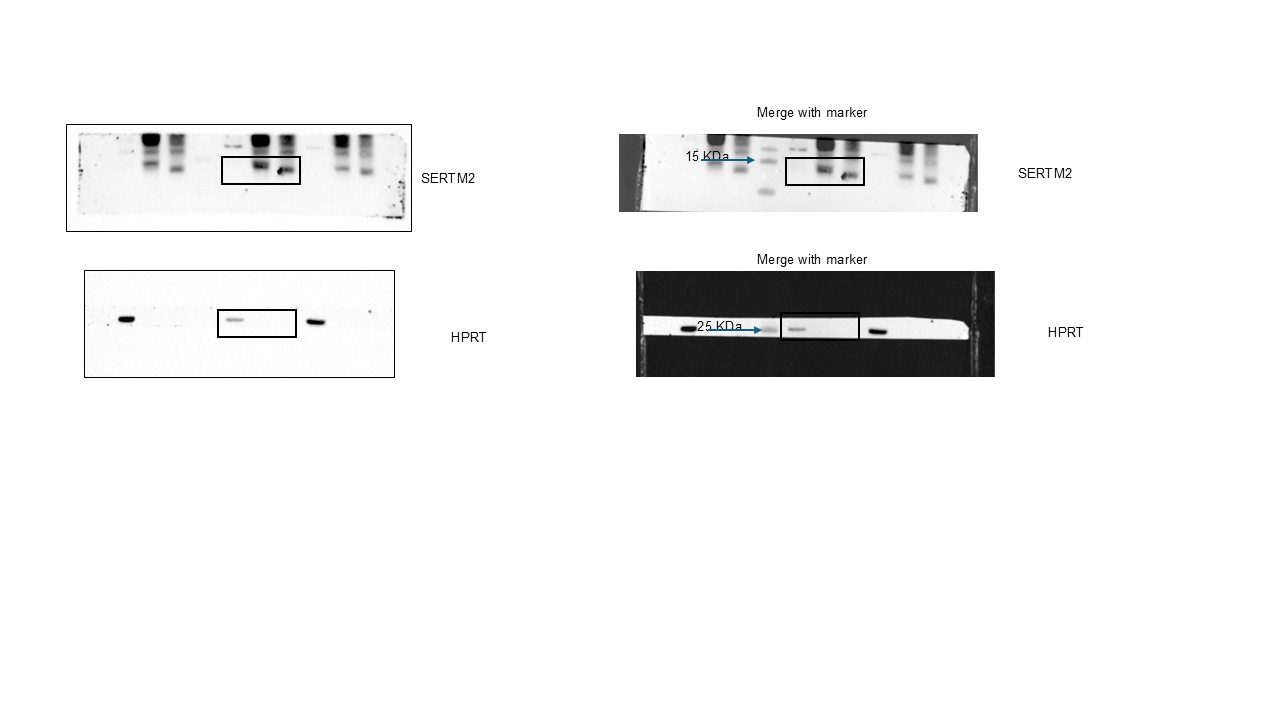

Supplement: Supplementary file 4 — Source data Fig. 2 [file 44319_2025_404_MOESM4_ESM.zip › Source data Figure 3/3K/3K.tif]

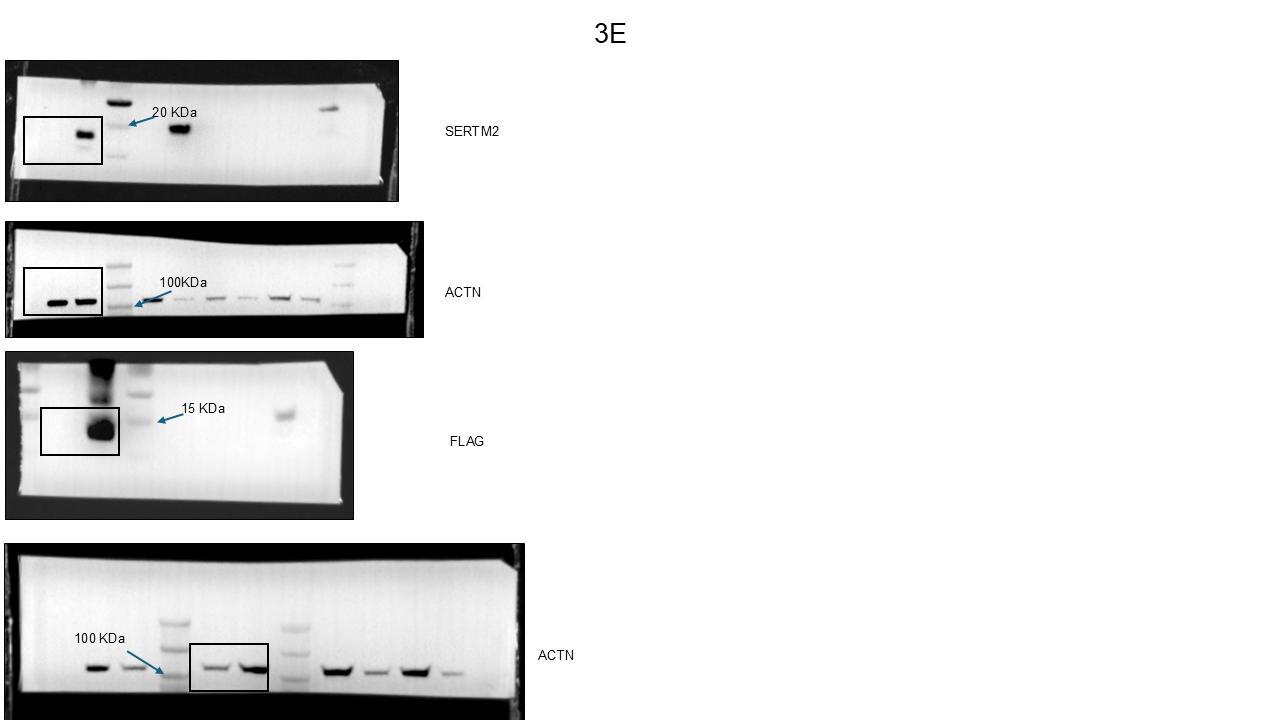

Supplement: Supplementary file 4 — Source data Fig. 2 [file 44319_2025_404_MOESM4_ESM.zip › Source data Figure 3/3E/3E.tif]

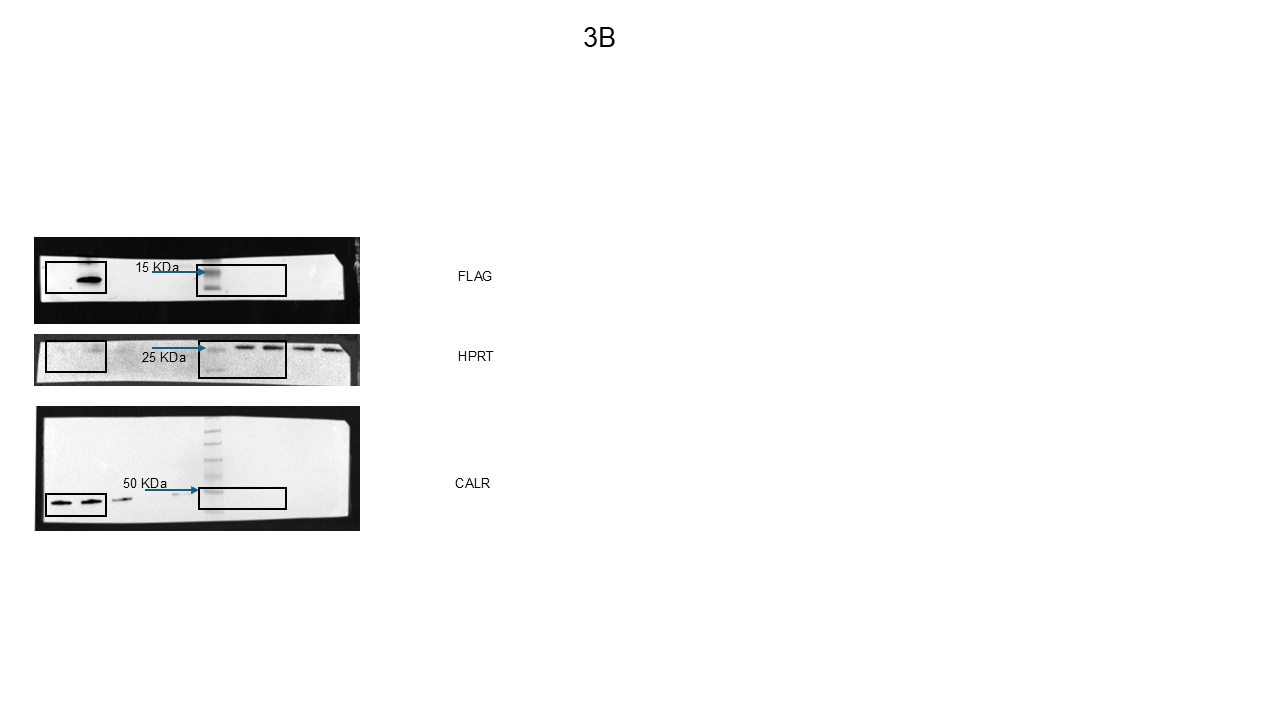

Supplement: Supplementary file 4 — Source data Fig. 2 [file 44319_2025_404_MOESM4_ESM.zip › Source data Figure 3/3B/3B.tif]

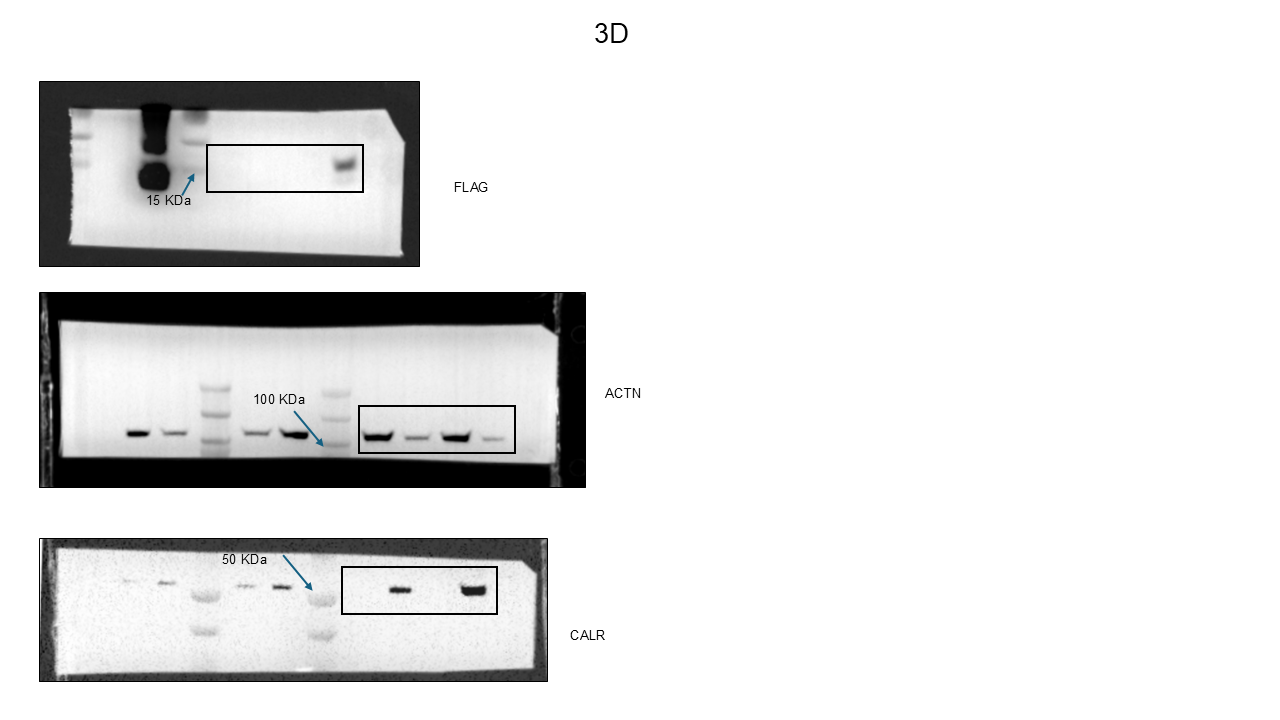

Supplement: Supplementary file 4 — Source data Fig. 2 [file 44319_2025_404_MOESM4_ESM.zip › Source data Figure 3/3D/3D.tif]

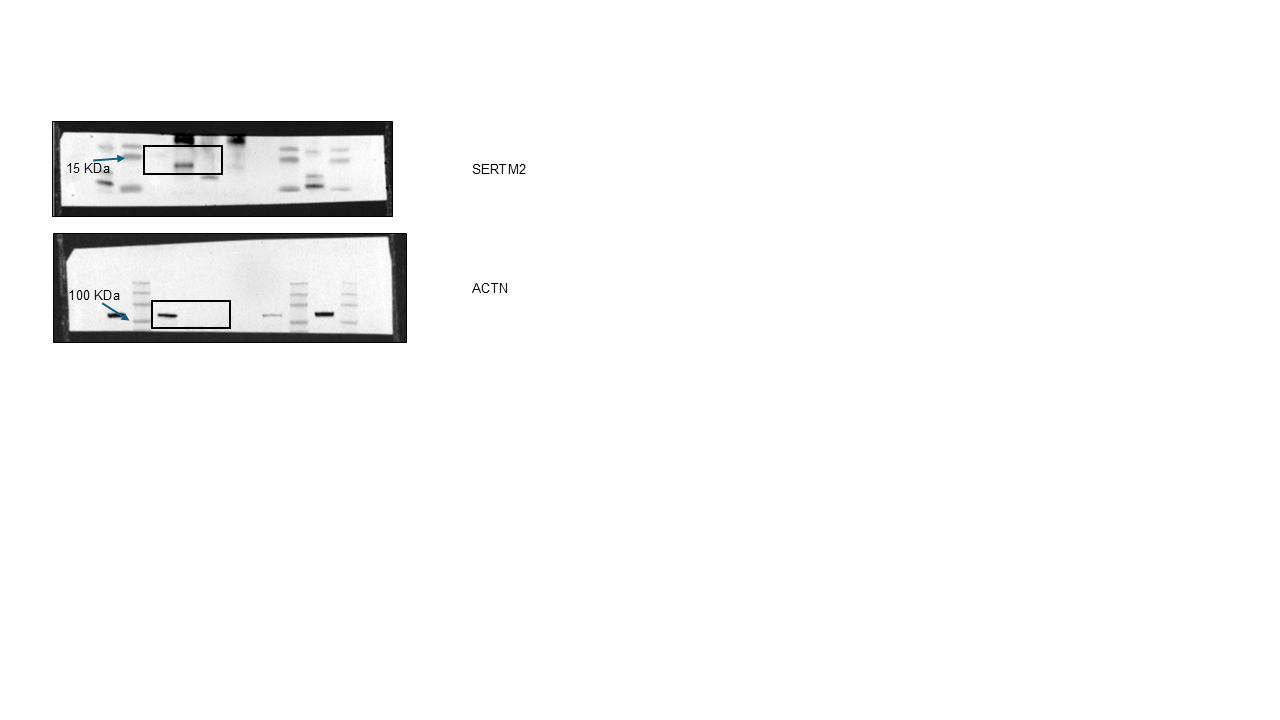

Supplement: Supplementary file 4 — Source data Fig. 2 [file 44319_2025_404_MOESM4_ESM.zip › Source data Figure 3/3J/3J.tif]

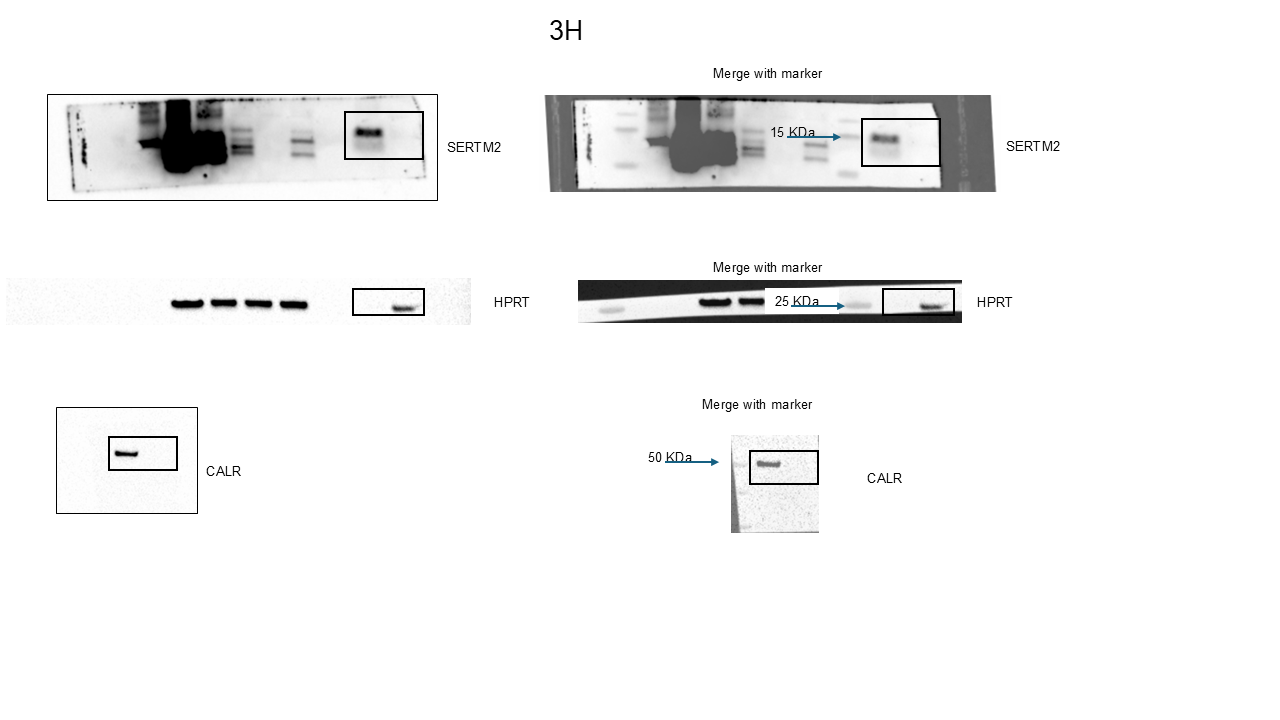

Supplement: Supplementary file 4 — Source data Fig. 2 [file 44319_2025_404_MOESM4_ESM.zip › Source data Figure 3/3H/3H.tif]

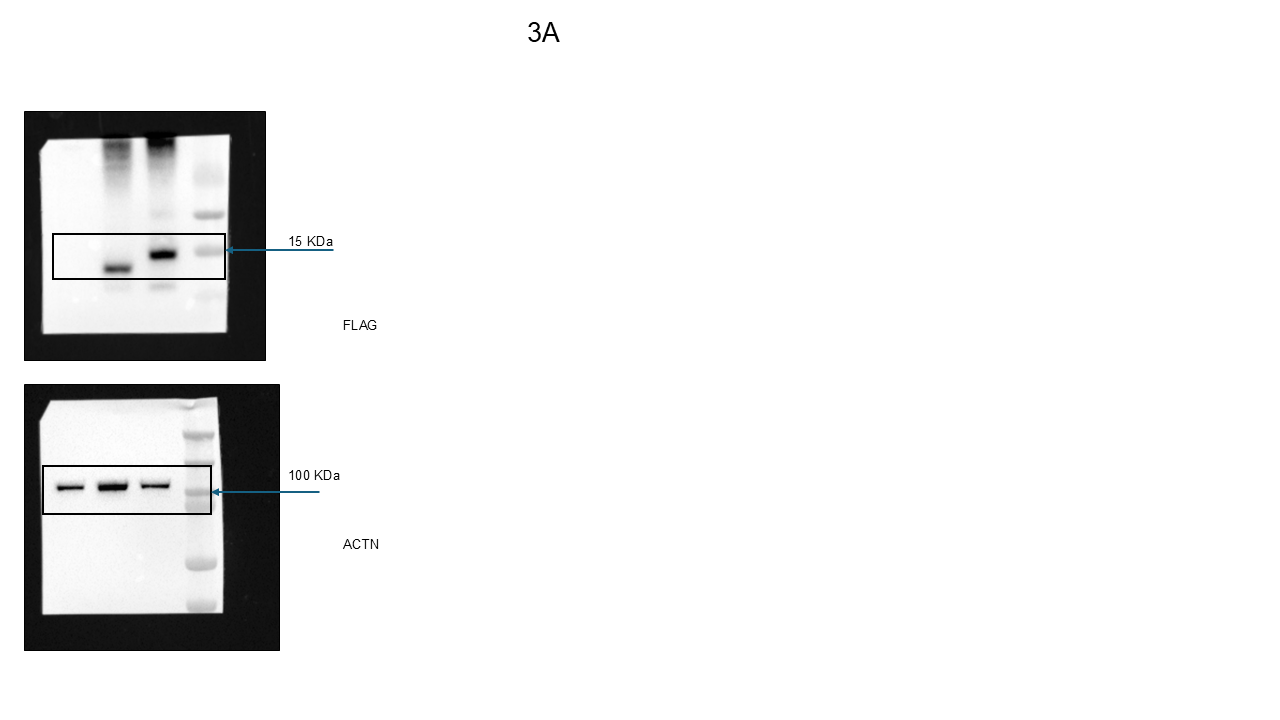

Supplement: Supplementary file 4 — Source data Fig. 2 [file 44319_2025_404_MOESM4_ESM.zip › Source data Figure 3/3A/3A.tif]

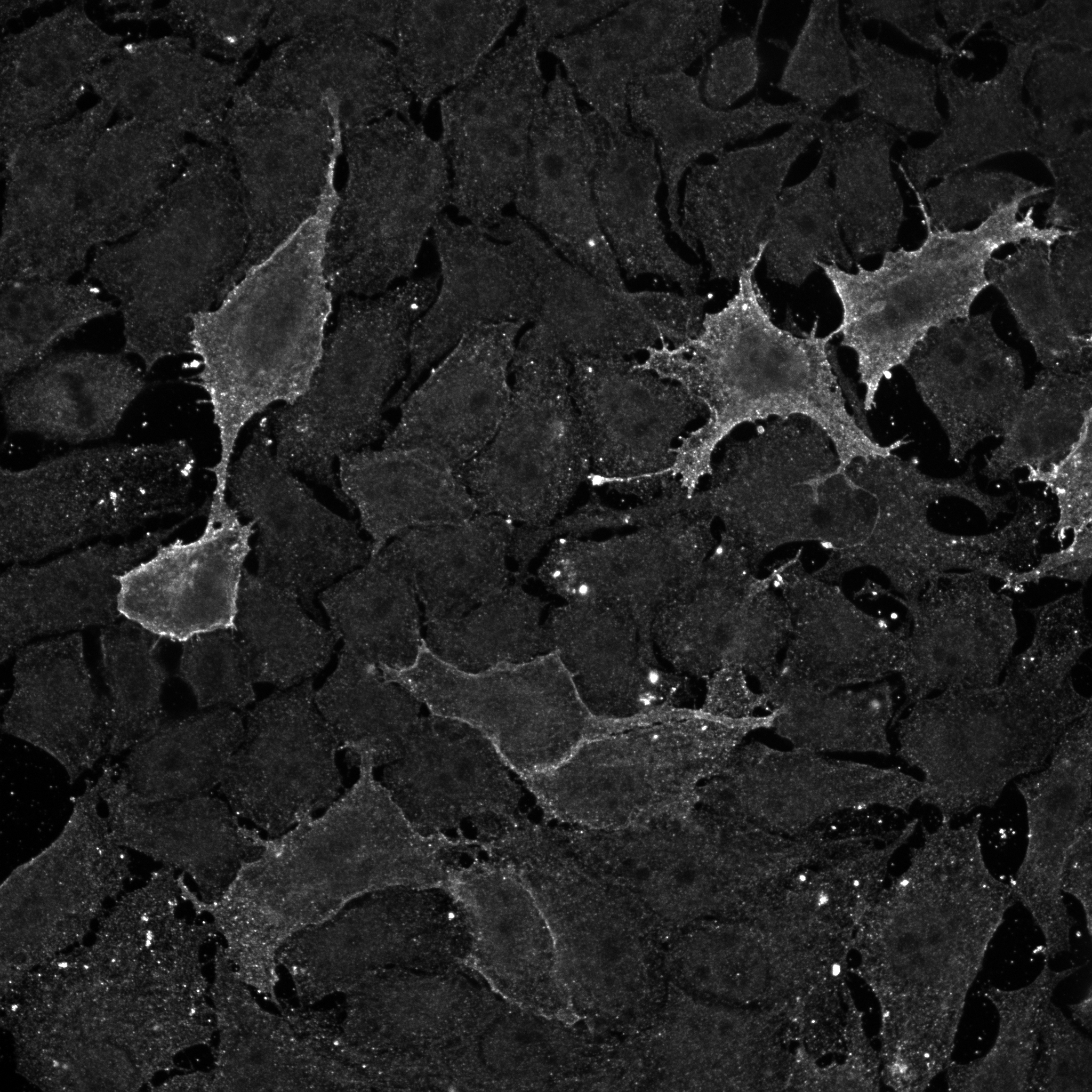

Supplement: Supplementary file 4 — Source data Fig. 2 [file 44319_2025_404_MOESM4_ESM.zip › Source data Figure 3/3F/FLAG-mSERTM2.tif]

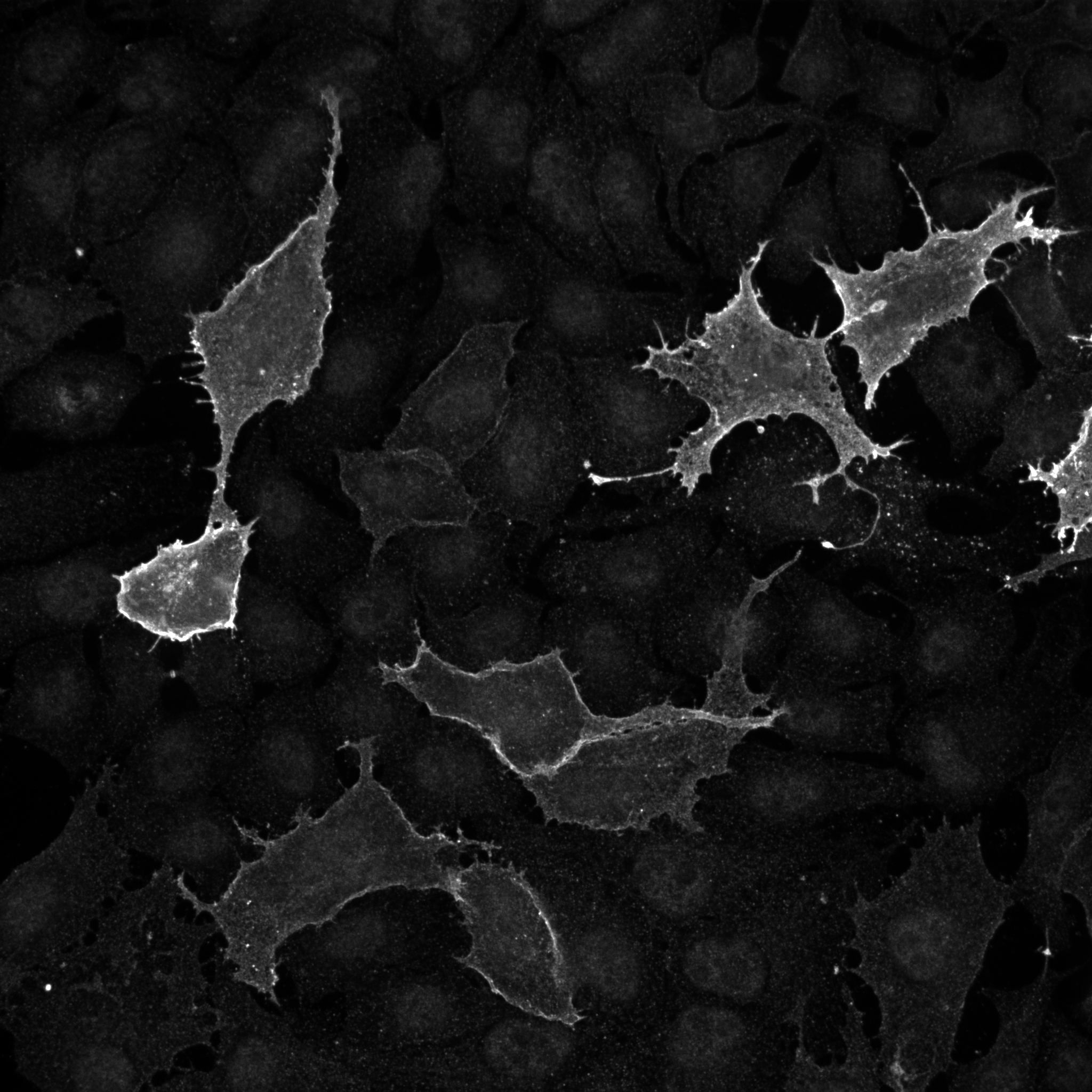

Supplement: Supplementary file 4 — Source data Fig. 2 [file 44319_2025_404_MOESM4_ESM.zip › Source data Figure 3/3F/mSERTM2.tif]

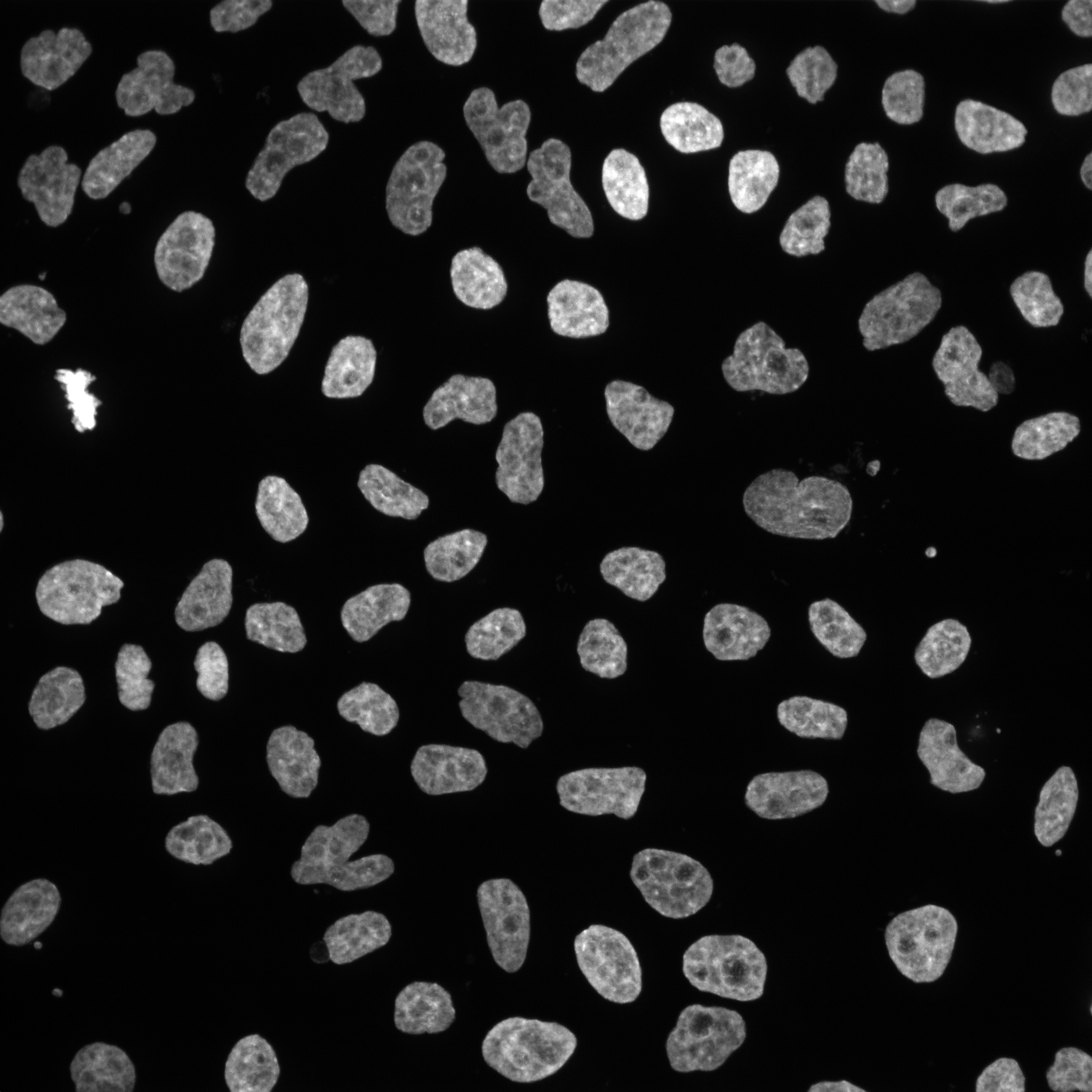

Supplement: Supplementary file 4 — Source data Fig. 2 [file 44319_2025_404_MOESM4_ESM.zip › Source data Figure 3/3F/DAPI.tif]

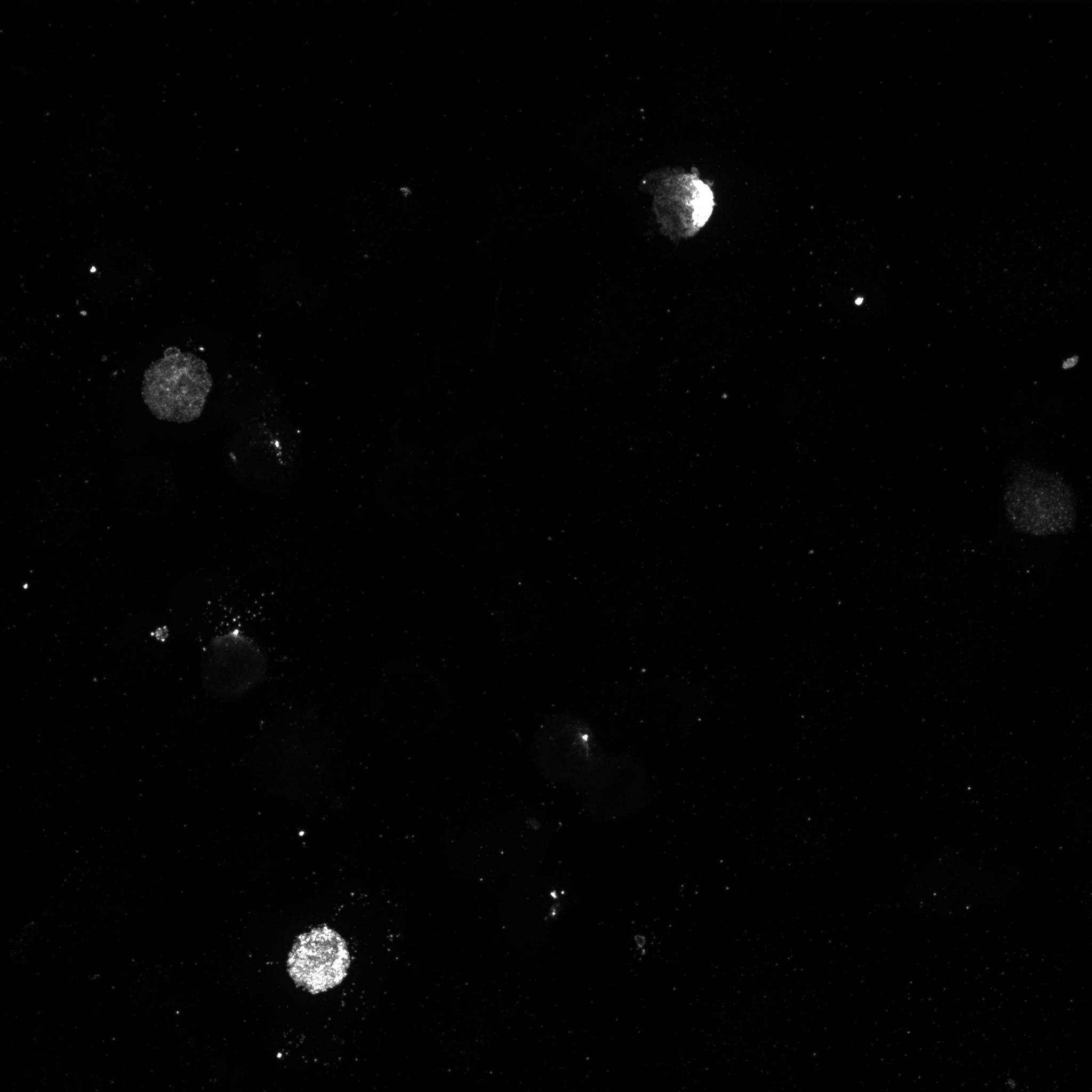

Supplement: Supplementary file 4 — Source data Fig. 2 [file 44319_2025_404_MOESM4_ESM.zip › Source data Figure 3/3G/mSERTM2.tif]

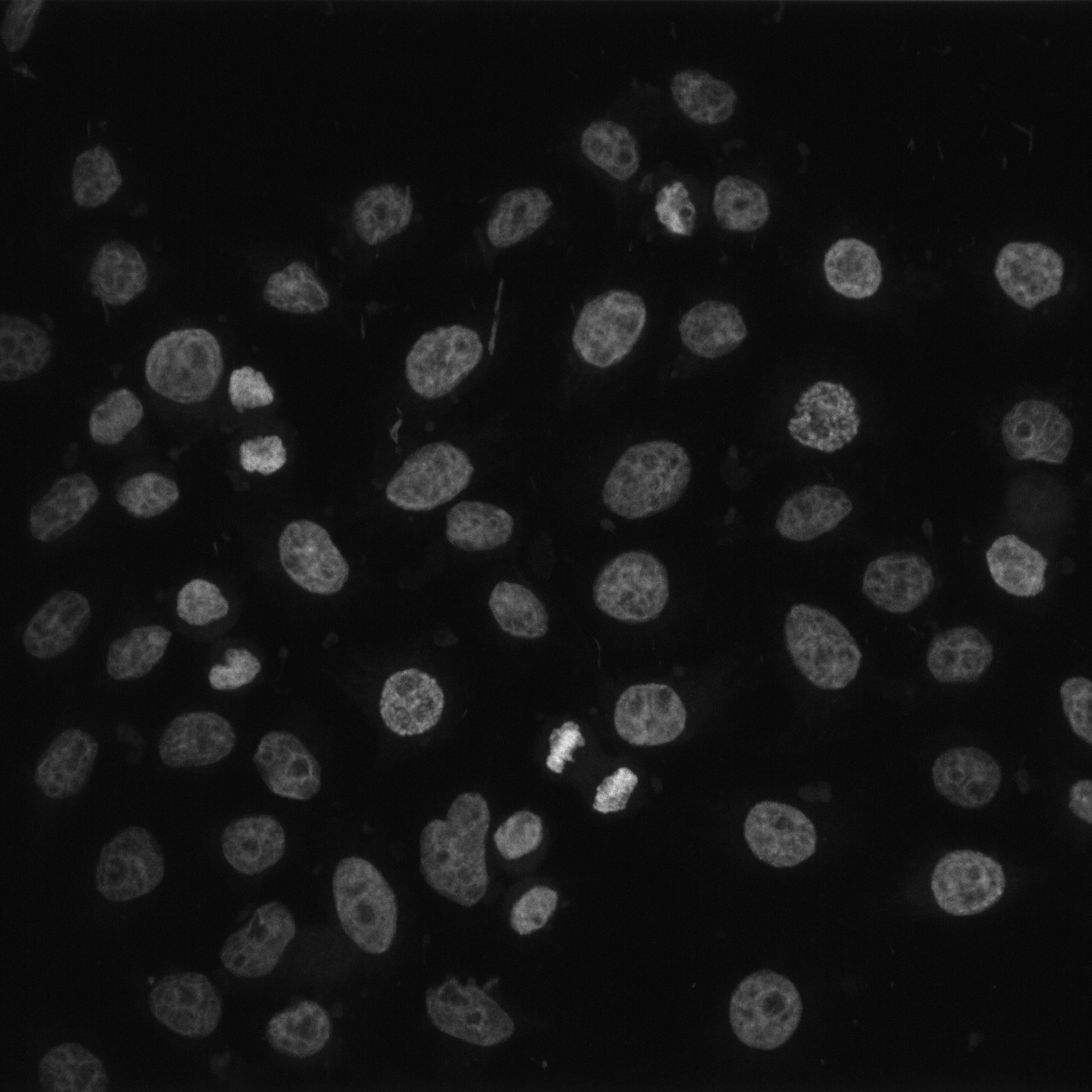

Supplement: Supplementary file 4 — Source data Fig. 2 [file 44319_2025_404_MOESM4_ESM.zip › Source data Figure 3/3G/DAPI.tif]

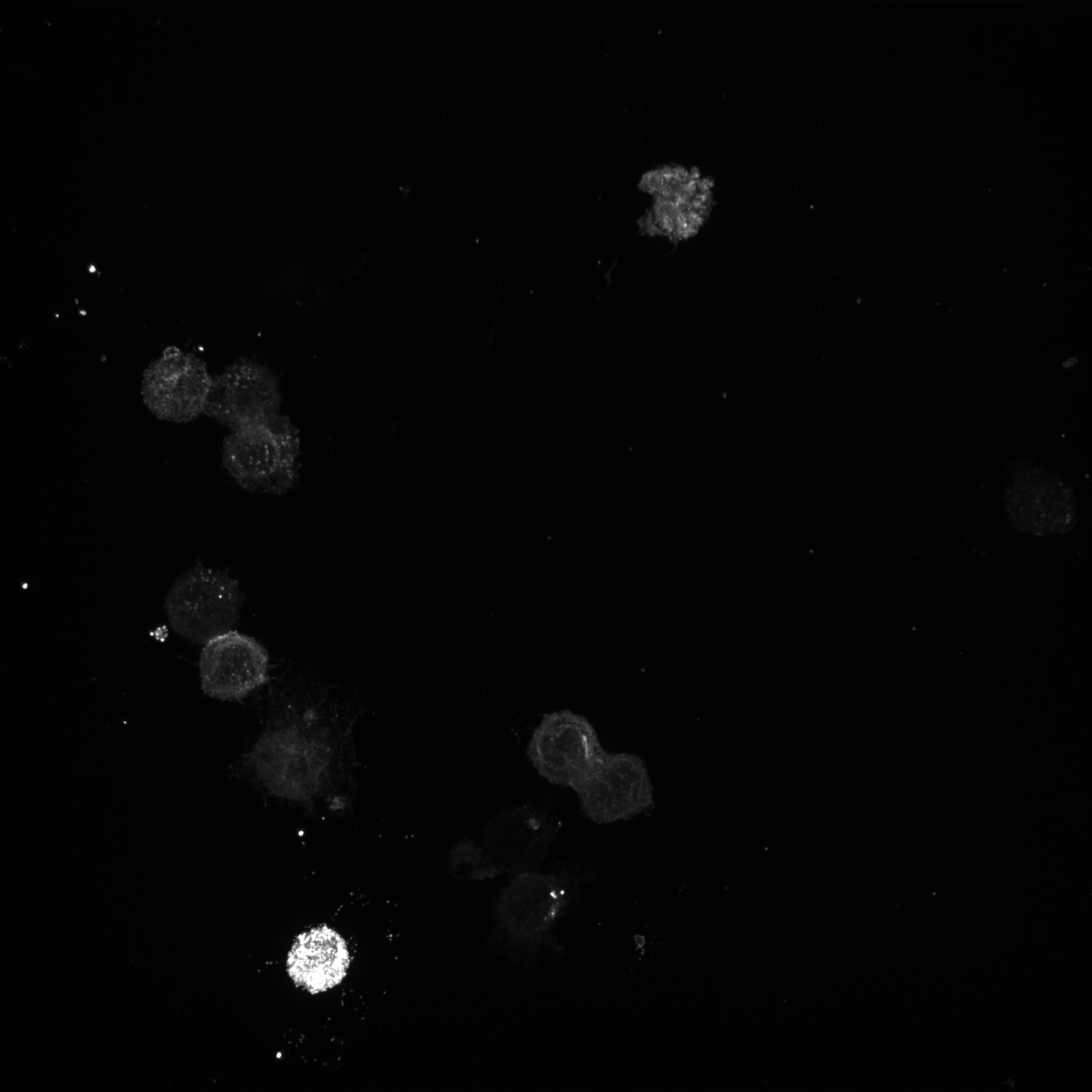

Supplement: Supplementary file 4 — Source data Fig. 2 [file 44319_2025_404_MOESM4_ESM.zip › Source data Figure 3/3G/FLAG-mSERTM2f.tif]

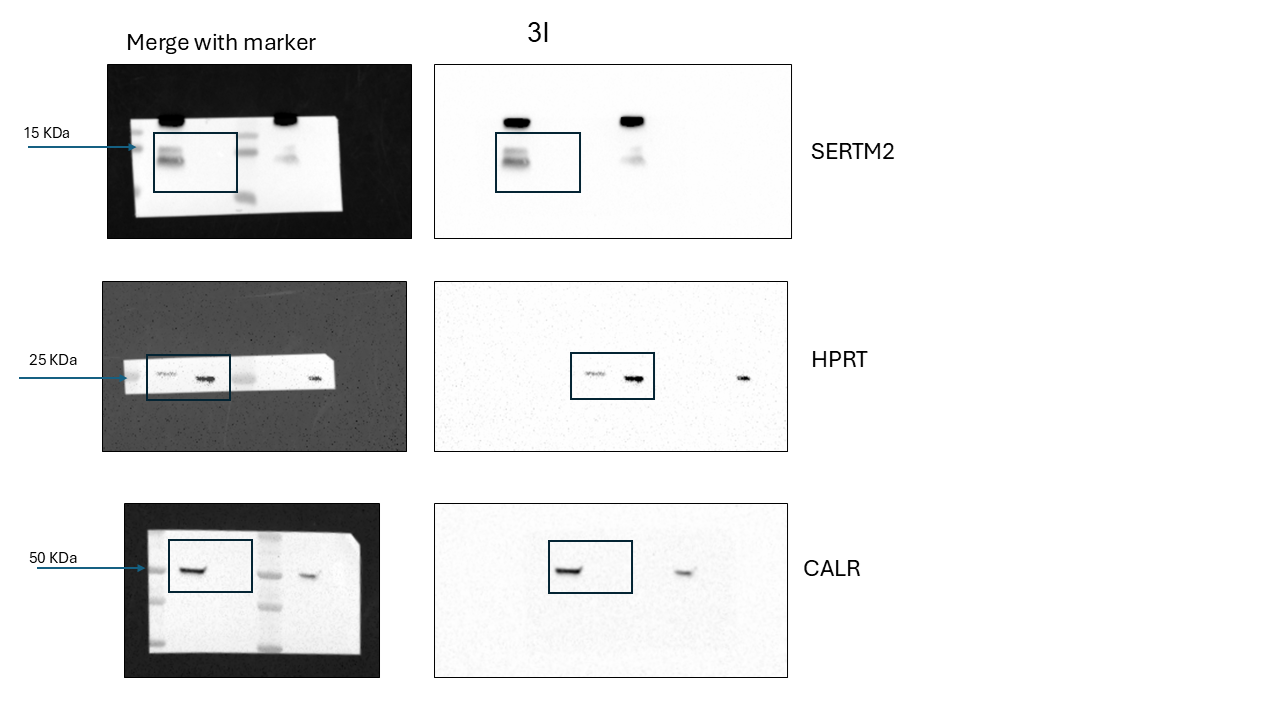

Supplement: Supplementary file 4 — Source data Fig. 2 [file 44319_2025_404_MOESM4_ESM.zip › Source data Figure 3/3I/3I .tif]

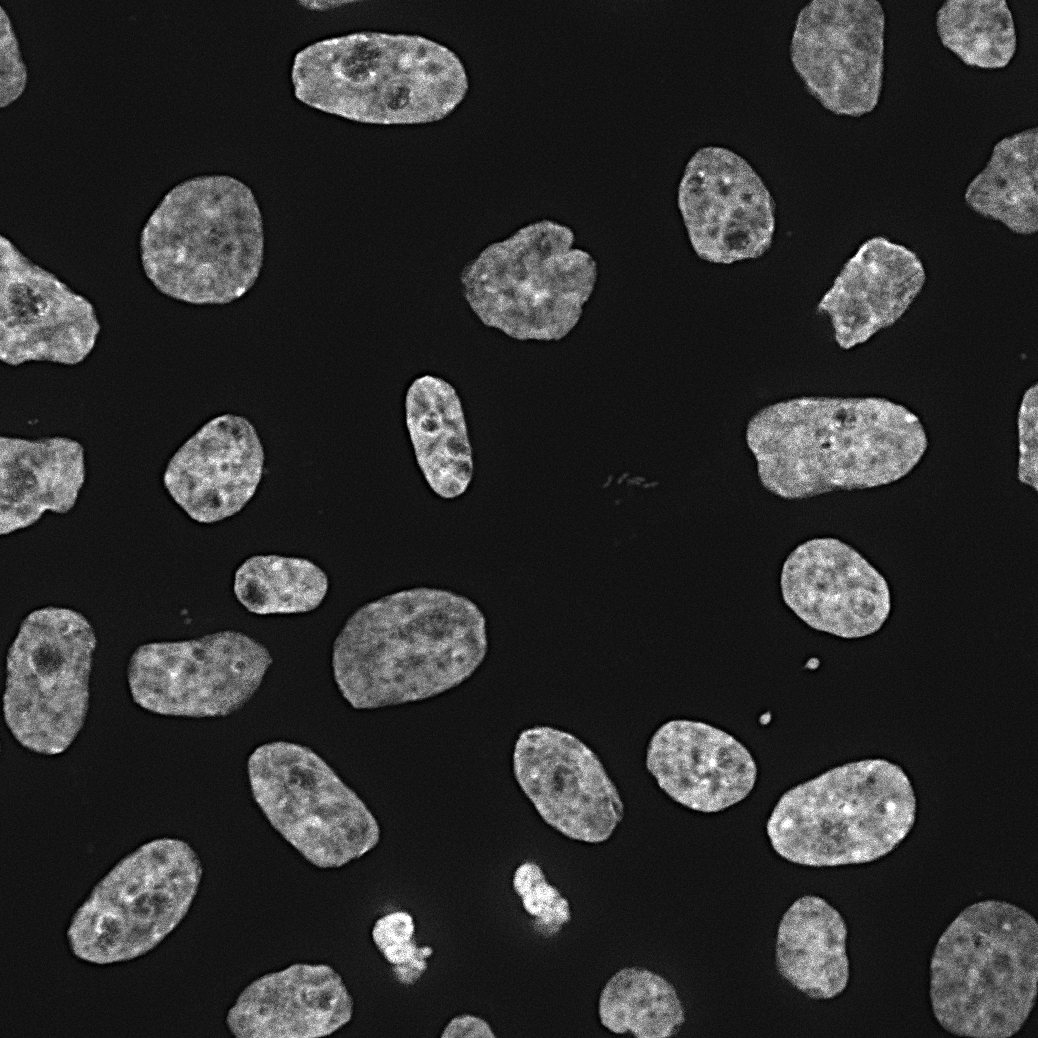

Supplement: Supplementary file 4 — Source data Fig. 2 [file 44319_2025_404_MOESM4_ESM.zip › Source data Figure 3/3C/FLAG/DAPI_magn.tif]

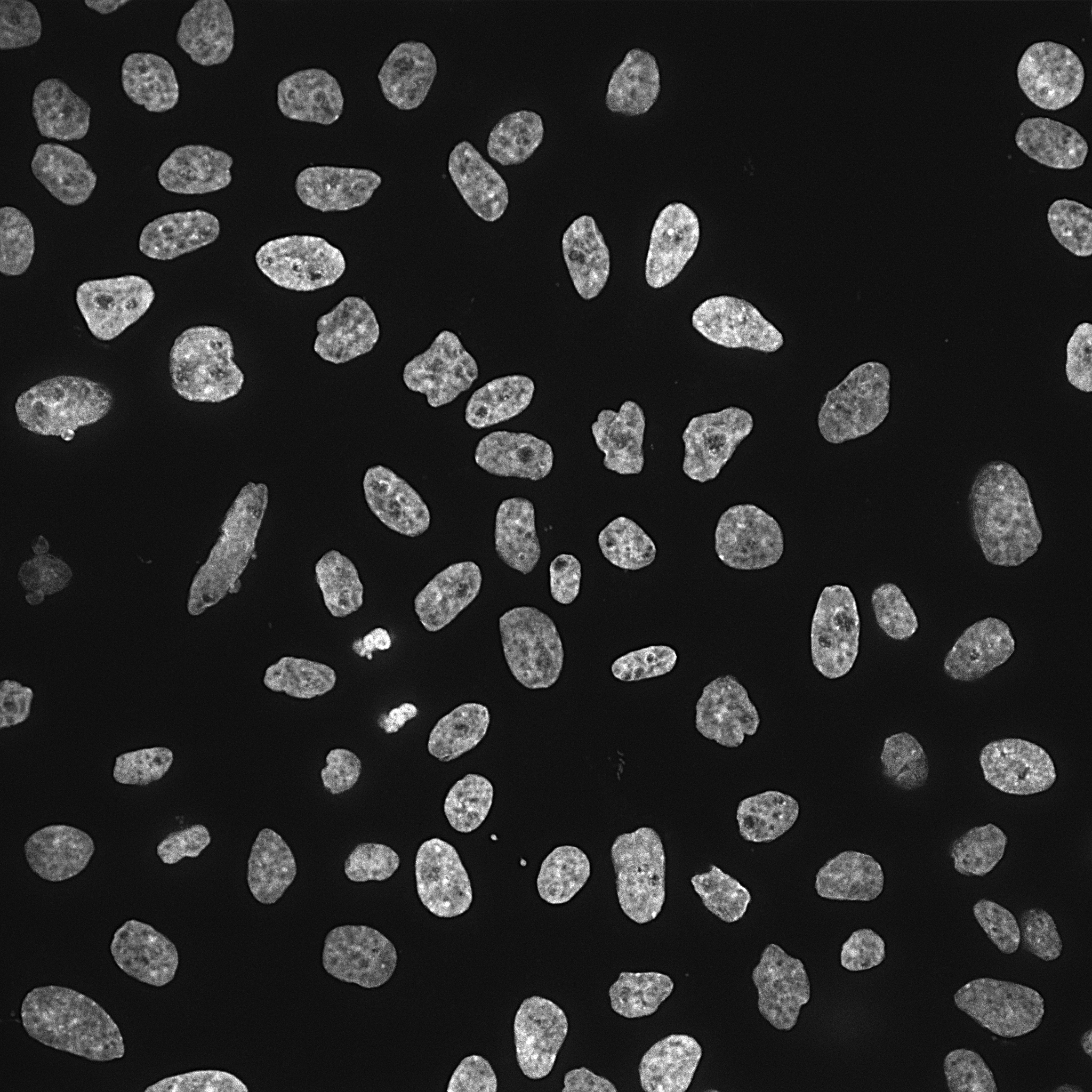

Supplement: Supplementary file 4 — Source data Fig. 2 [file 44319_2025_404_MOESM4_ESM.zip › Source data Figure 3/3C/FLAG/DAPI_full.tif]

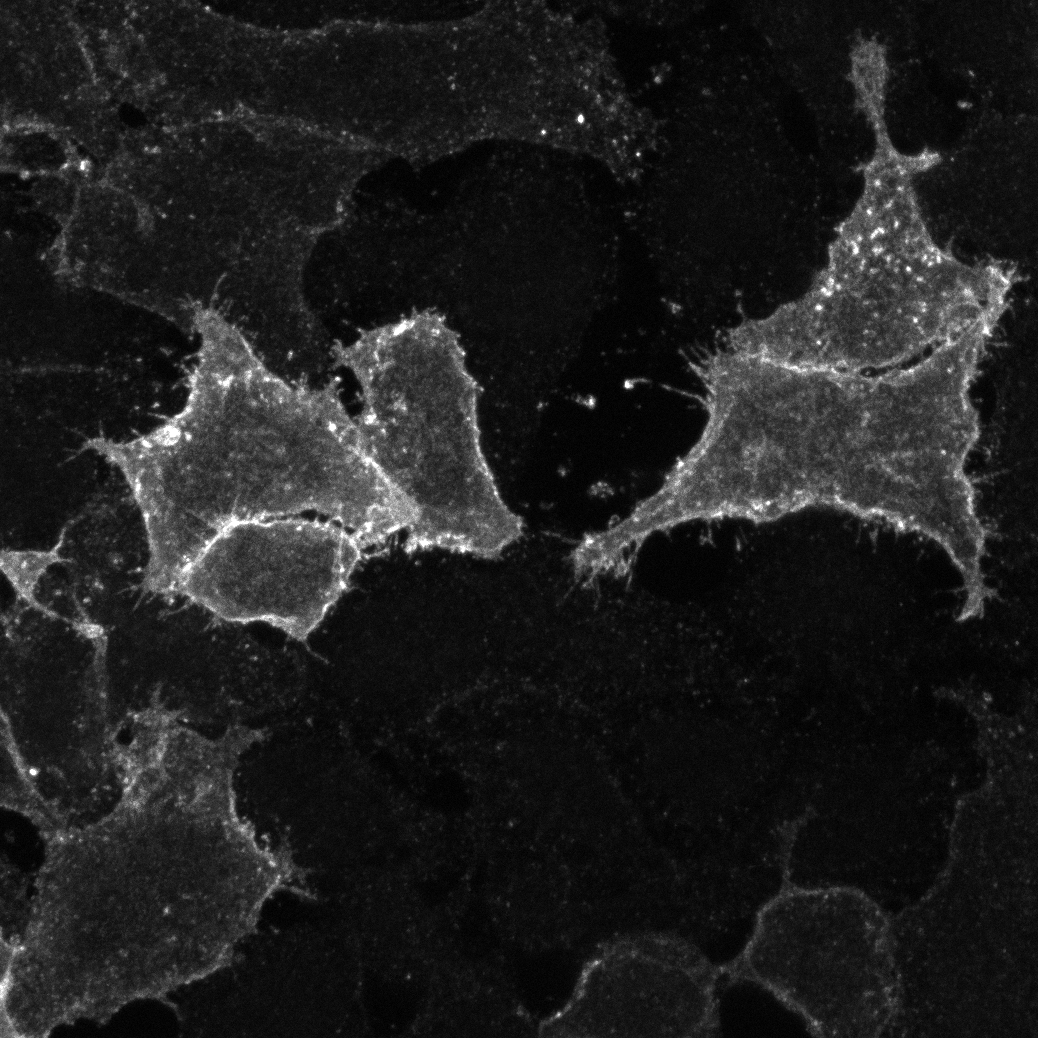

Supplement: Supplementary file 4 — Source data Fig. 2 [file 44319_2025_404_MOESM4_ESM.zip › Source data Figure 3/3C/FLAG/FLAG-mSERTM2_magn.tif]

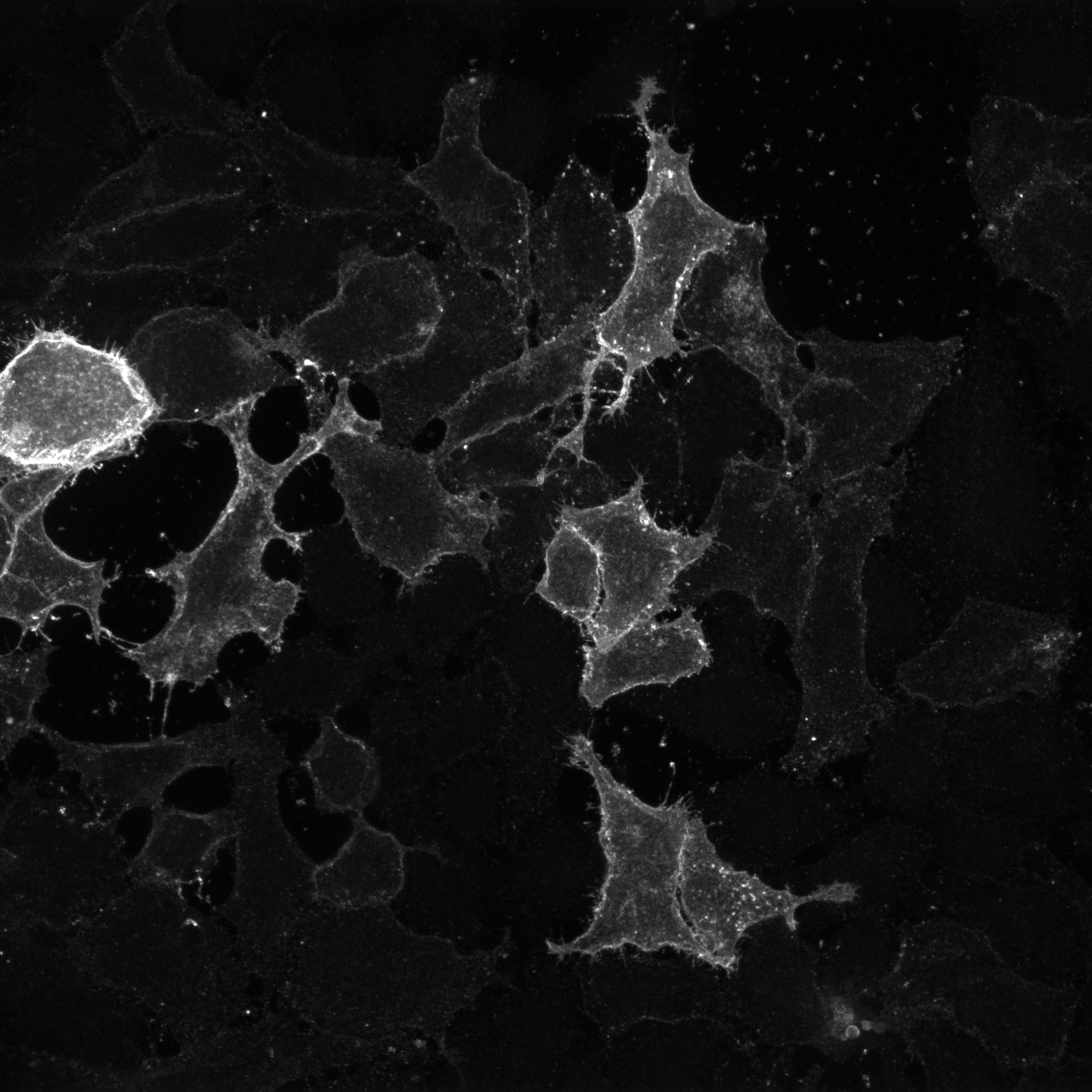

Supplement: Supplementary file 4 — Source data Fig. 2 [file 44319_2025_404_MOESM4_ESM.zip › Source data Figure 3/3C/FLAG/FLAG-mSERTM2_full.tif]

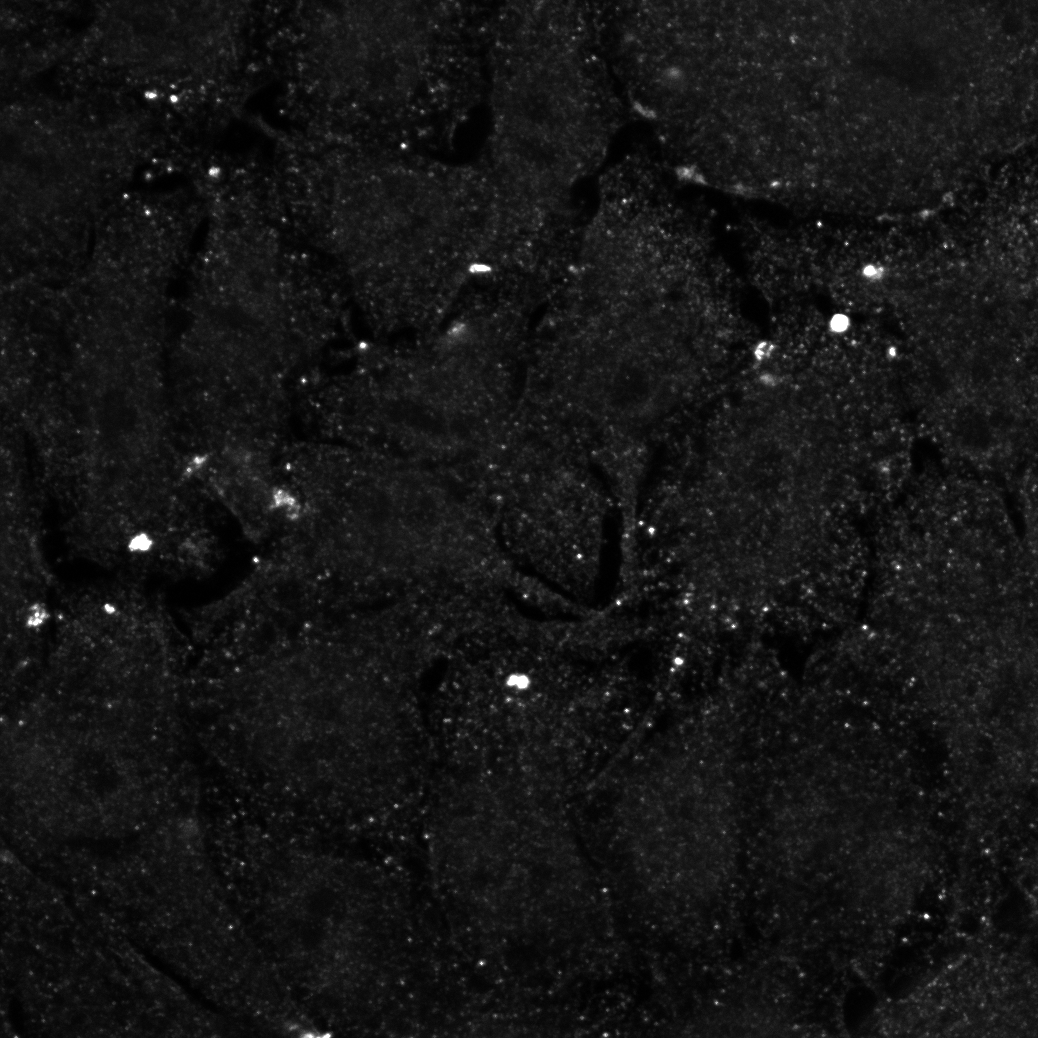

Supplement: Supplementary file 4 — Source data Fig. 2 [file 44319_2025_404_MOESM4_ESM.zip › Source data Figure 3/3C/pcDNA/pcDNA FLAG_ magn.tif]

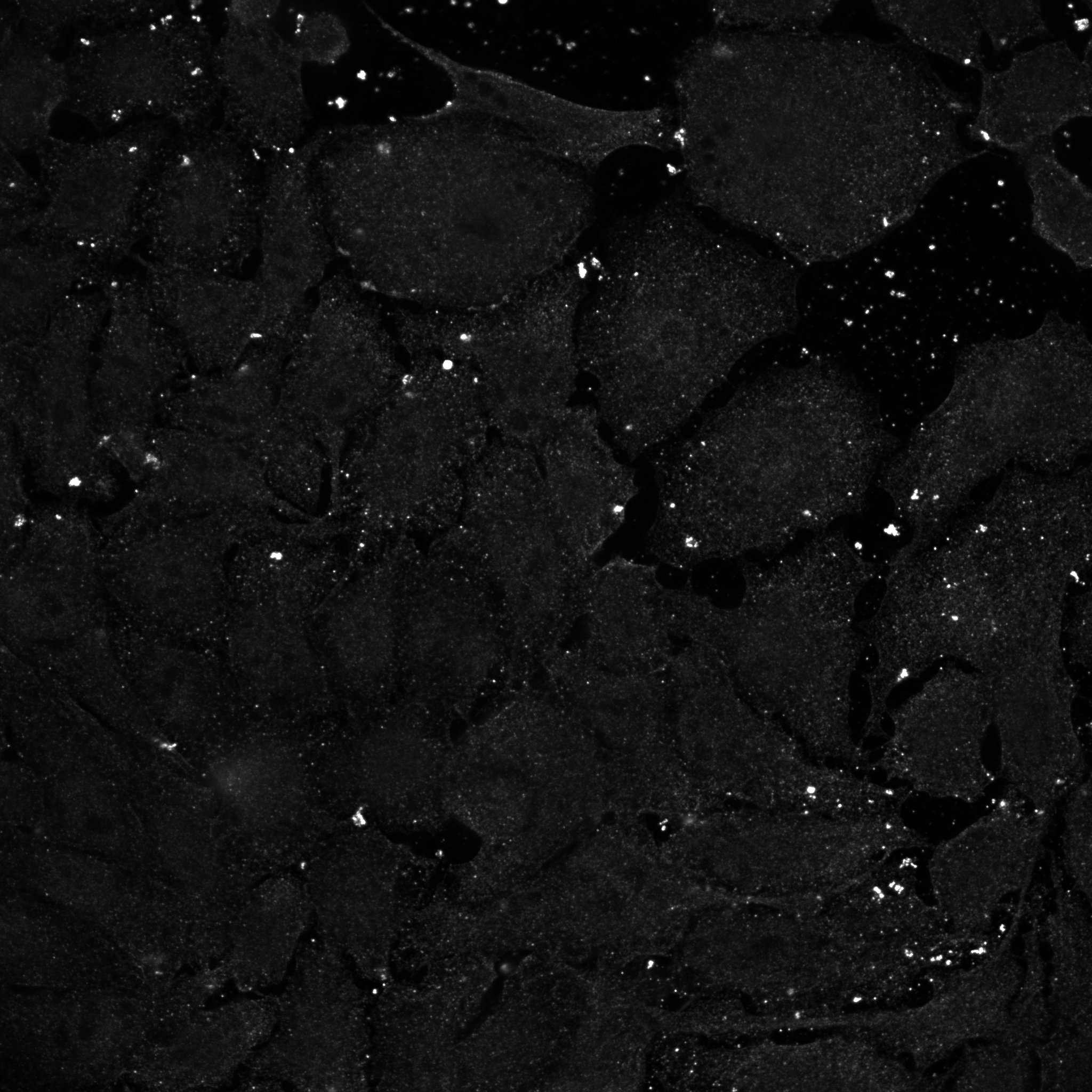

Supplement: Supplementary file 4 — Source data Fig. 2 [file 44319_2025_404_MOESM4_ESM.zip › Source data Figure 3/3C/pcDNA/pcDNA FLAG_full .tif]

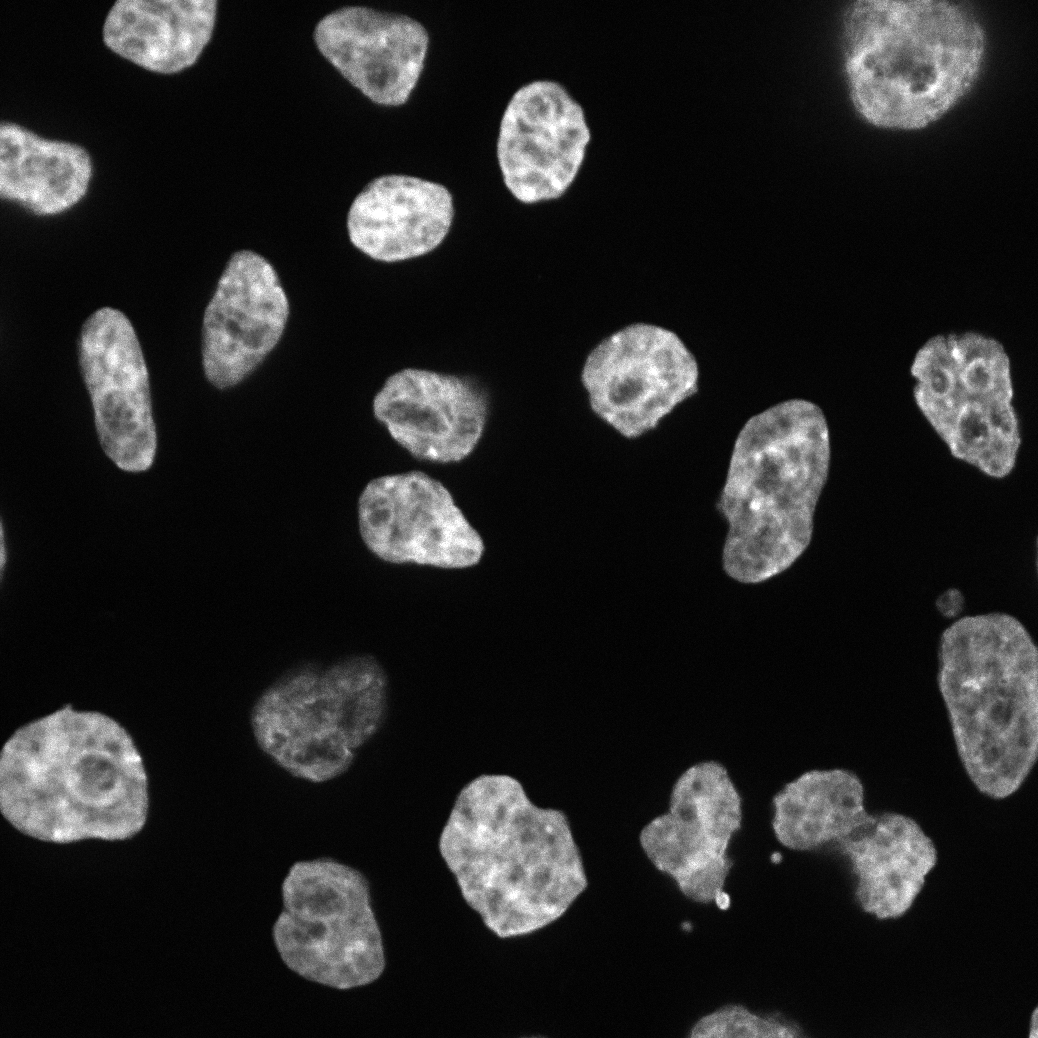

Supplement: Supplementary file 4 — Source data Fig. 2 [file 44319_2025_404_MOESM4_ESM.zip › Source data Figure 3/3C/pcDNA/DAPI_magn.tif]

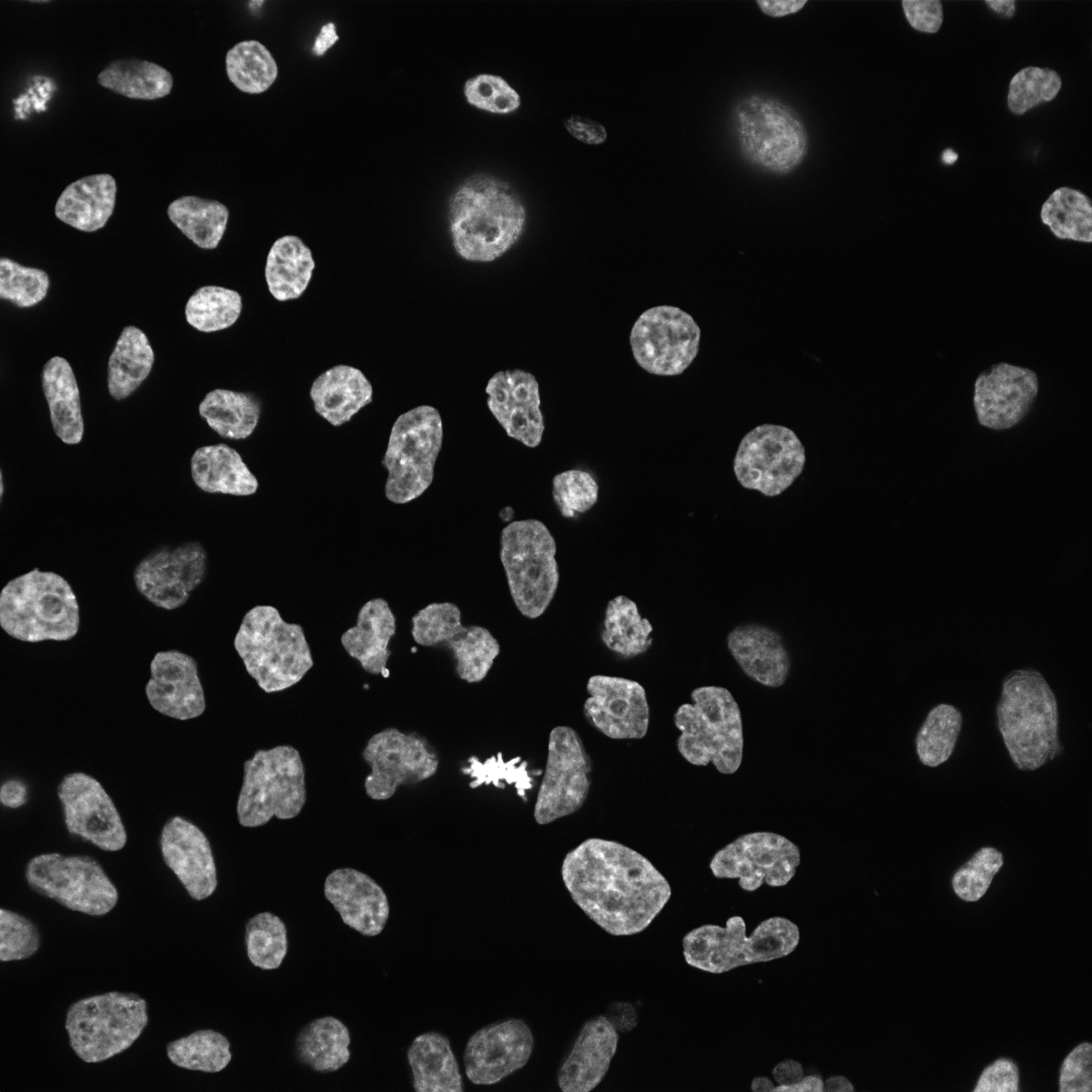

Supplement: Supplementary file 4 — Source data Fig. 2 [file 44319_2025_404_MOESM4_ESM.zip › Source data Figure 3/3C/pcDNA/DAPI_full.tif]

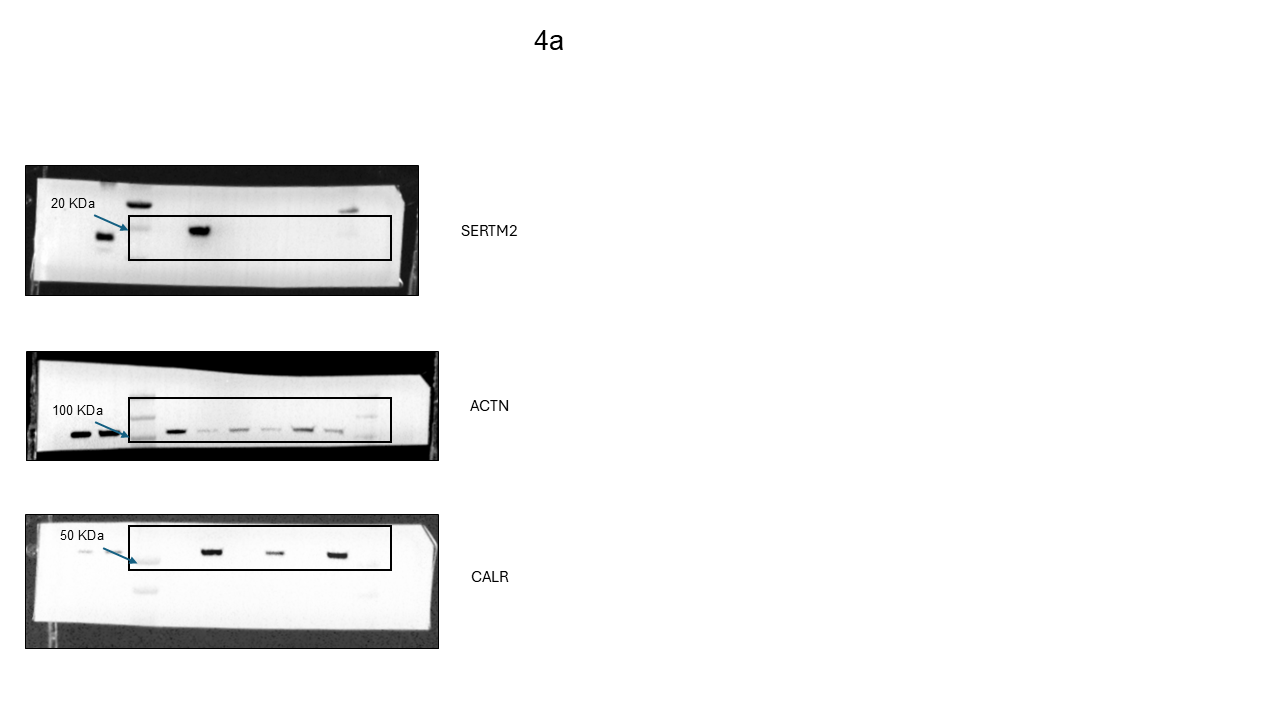

Supplement: Supplementary file 6 — Source data Fig. 4 [file 44319_2025_404_MOESM6_ESM.zip › Source data Figure 4/4A/4A.tif]

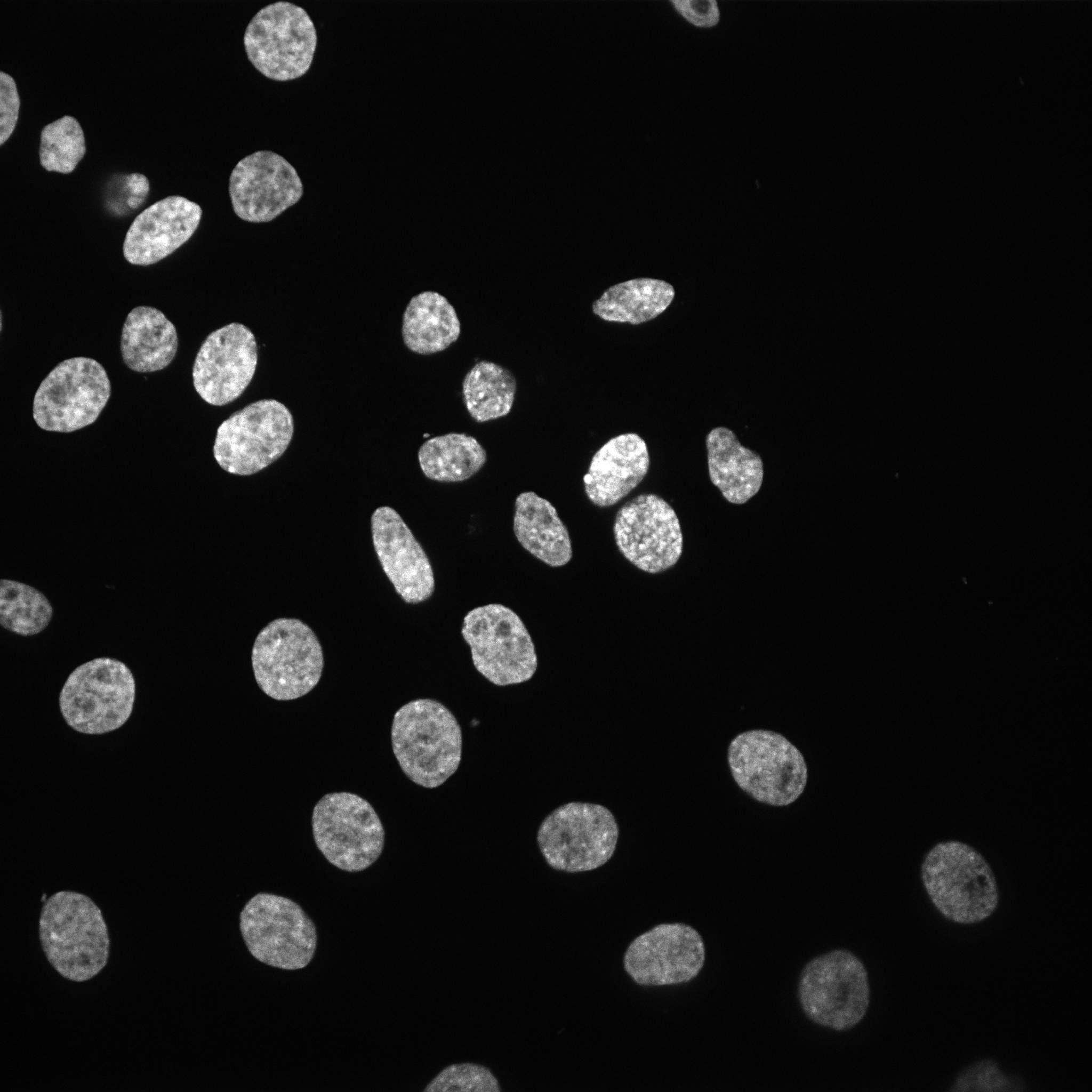

Supplement: Supplementary file 7 — Source data Fig. 5 [file 44319_2025_404_MOESM7_ESM.zip › Source data Figure 5/5 E_F/DAPI.tif]

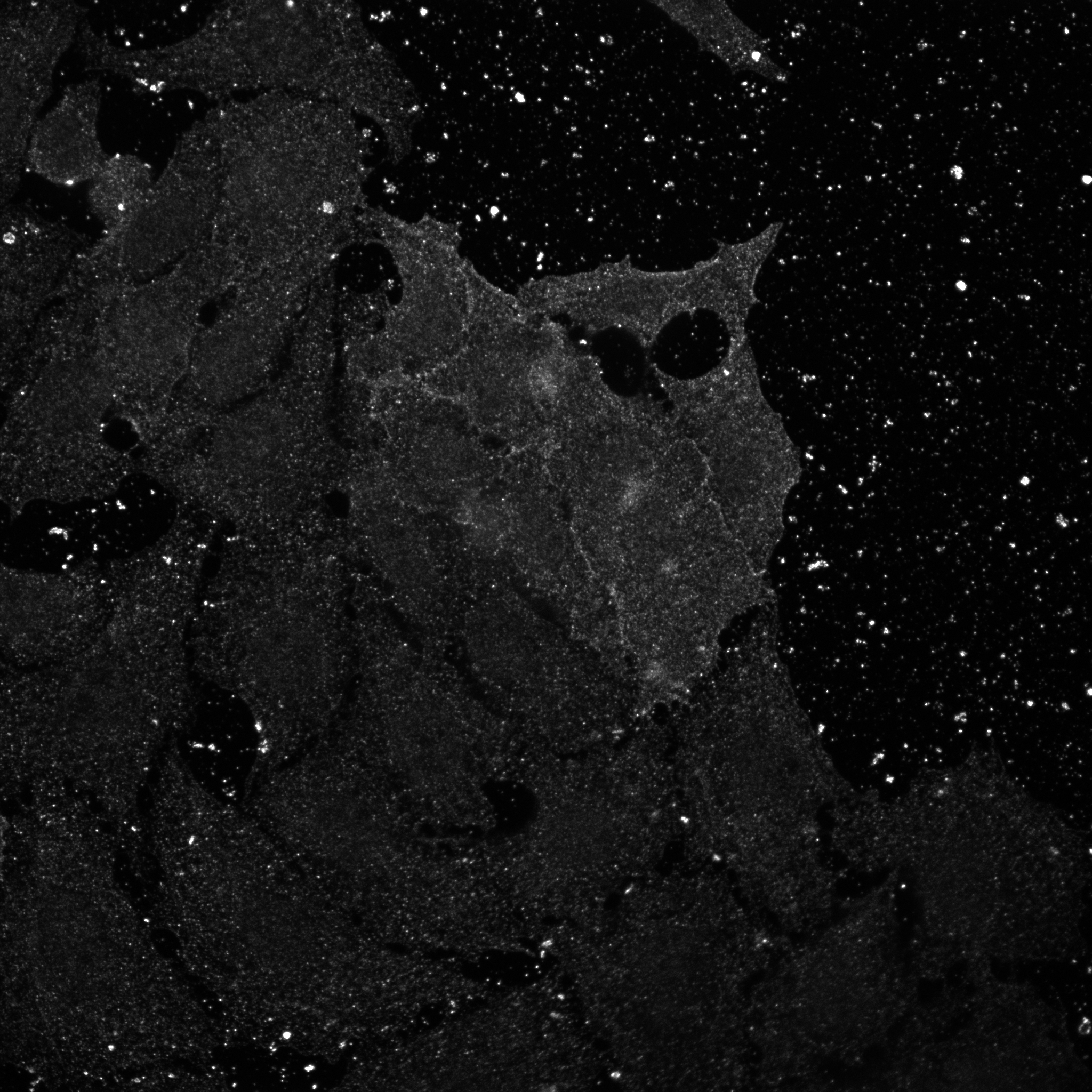

Supplement: Supplementary file 7 — Source data Fig. 5 [file 44319_2025_404_MOESM7_ESM.zip › Source data Figure 5/5 E_F/FLAG-mSERTM2.tif]

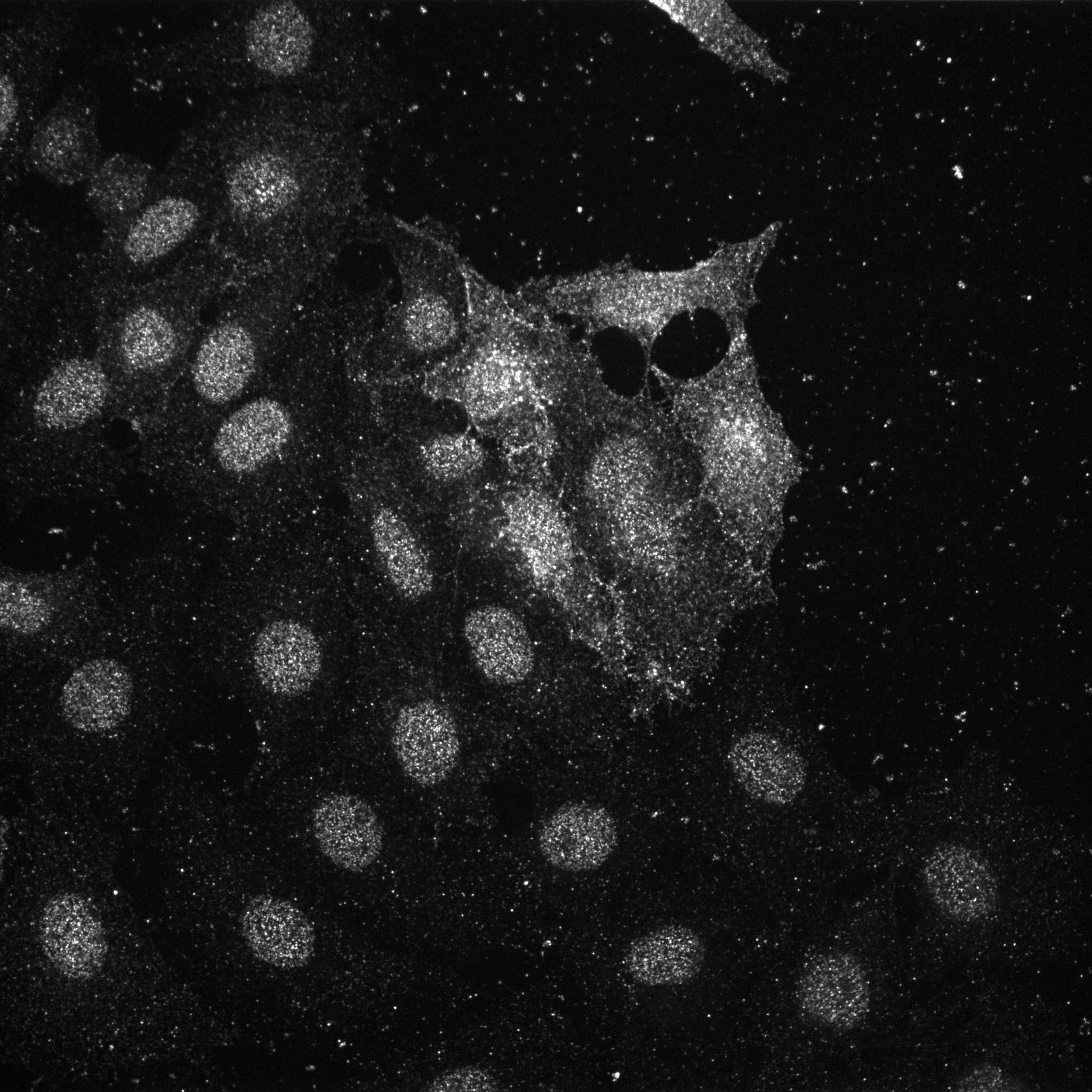

Supplement: Supplementary file 7 — Source data Fig. 5 [file 44319_2025_404_MOESM7_ESM.zip › Source data Figure 5/5 E_F/Task1.tif]

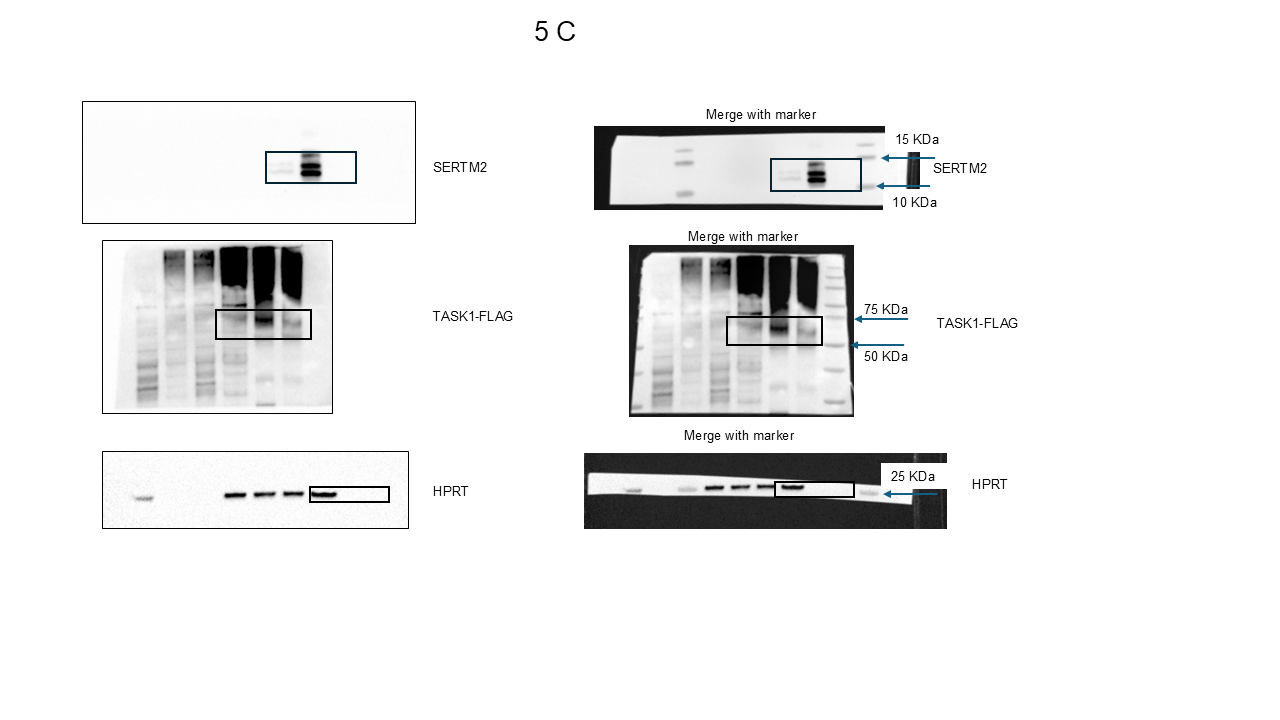

Supplement: Supplementary file 7 — Source data Fig. 5 [file 44319_2025_404_MOESM7_ESM.zip › Source data Figure 5/5C/5C.tif]

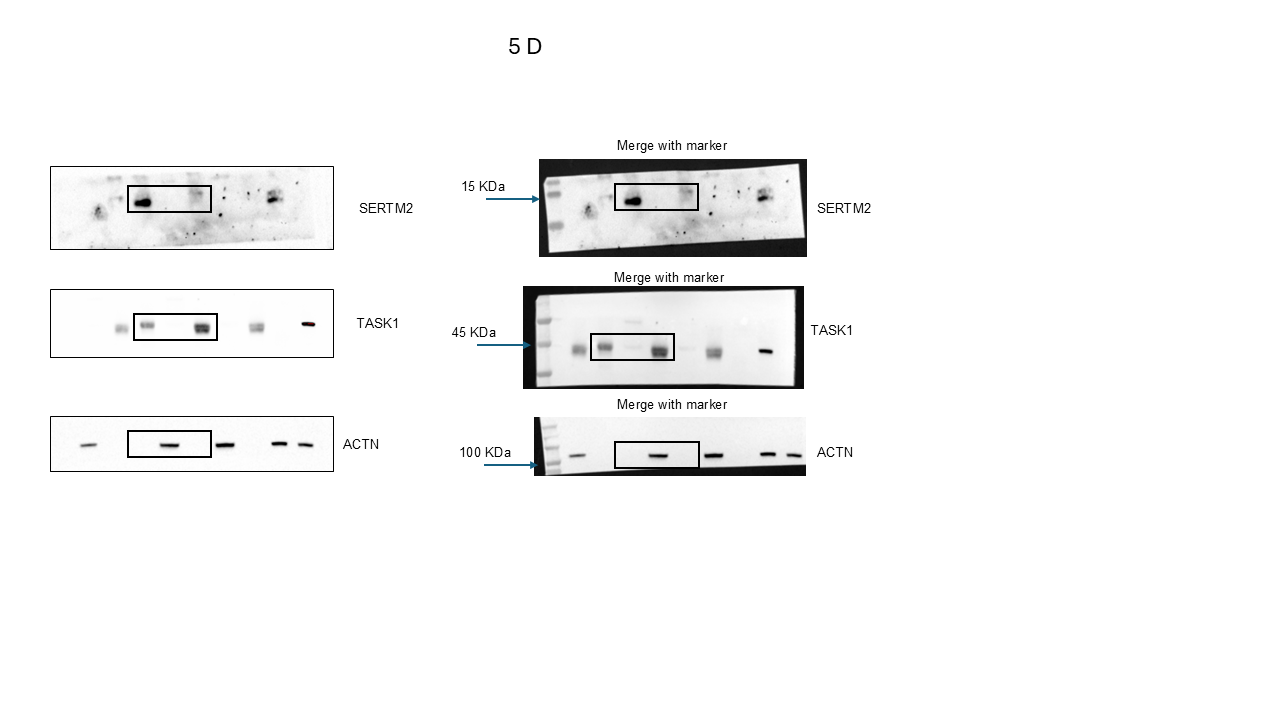

Supplement: Supplementary file 7 — Source data Fig. 5 [file 44319_2025_404_MOESM7_ESM.zip › Source data Figure 5/5D/5D.tif]

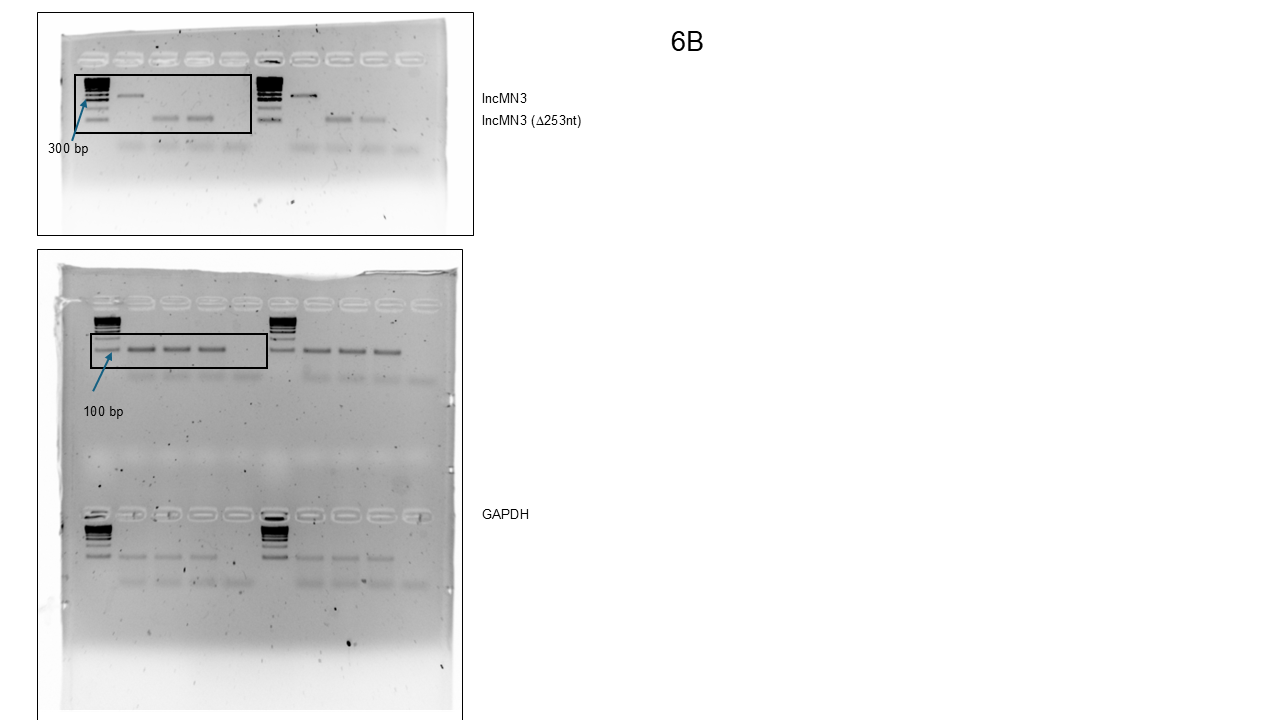

Supplement: Supplementary file 8 — Source data Fig. 6 [file 44319_2025_404_MOESM8_ESM.zip › Source data Figure 6/6B/6B.tif]

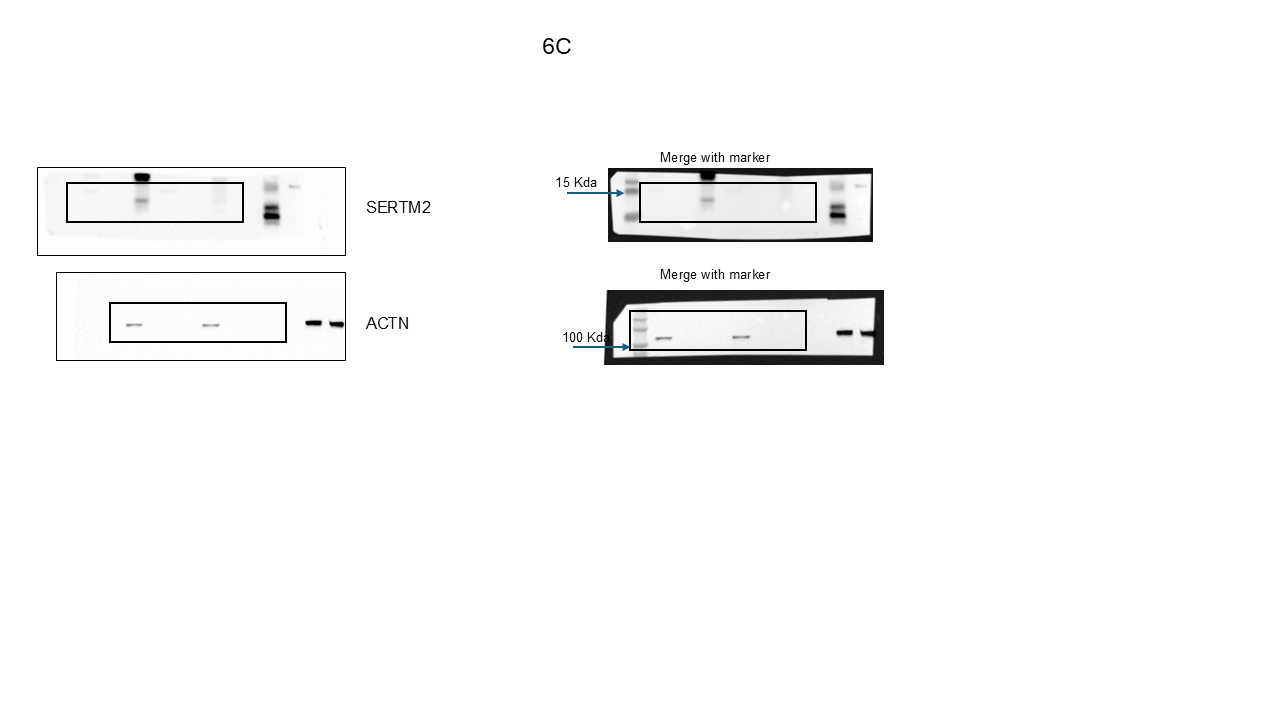

Supplement: Supplementary file 8 — Source data Fig. 6 [file 44319_2025_404_MOESM8_ESM.zip › Source data Figure 6/6C/6C.tif]
