# Supplementary material for: SERTM2: a neuroactive player in the world of micropeptides
Source: EMBO Rep. 2025 Mar 19;26(8):2044–76. doi: 10.1038/s44319-025-00404-w (PMC12019361; doi:10.1038/s44319-025-00404-w)
Supplement: Supplementary file 9 — Expanded View Figures [file 44319_2025_404_MOESM9_ESM.pdf]

Expanded View Figures

Figure EV1. Characterization of lncMN3 expression profile.

(**A**) UMAP plot of the integrated dataset from Carvelli et al (2021) depicting cell identity assignment to NP, MNP, EMN, LMN, IN, or NA (cells that could not be assigned to any specific identity) subpopulations. (**B**) Single-cell expression of lncMN3 and marker genes over the UMAP representation of the integrated dataset. (**C**) qRT-PCR showing the expression levels of lncMN3 in spinal cord RNA samples obtained from mice at the indicated ages. The expression levels were normalized against the GAPDH mRNA and expressed as relative fold change with respect to a P3 sample set to a value of 1. The mean ± SD of 3 mice for each group is shown. (**D**) Left panel: UMAP visualization of single-cell RNA sequencing data from mouse spinal cord. Cell types of interest are color-coded according to their identity: orange for progenitor of motor neurons (pMN) and violet for motor neurons (MN). Middle panel: Expression levels of lncMN3 (A730046J19Rik) in the UMAP plot derived from mouse spinal cord single-cell RNA sequencing. lncMN3 expression levels are represented with violet-yellow color scale: higher intensity (yellow) corresponds to higher expression levels, while lower intensity (violet) indicates lower expression levels. Right panel: Dotplot depicting lncMN3 (A730046J19Rik) expression levels in mouse spinal cord cell populations. Y-axis depicts identified cell populations while x-axis depicts for each cell population the fraction of cells expressing the gene. LncMN3 expression levels are represented with yellow-red color scale: higher intensity (dark red) corresponds to higher expression levels, while lower intensity (yellow) indicates lower expression levels. The expression of lncMN3 in MN is highlighted by the orange box. (**E**) Left panel: UMAP visualization of single-cell RNA sequencing data from human spinal cord. Cell types of interest are color-coded according to their identity: lilac for progenitor of motor neurons (pMN) and green for motor neurons (MN). Middle panel: Expression levels of lncMN3 (SERTM2) in the UMAP plot derived from human spinal cord single-cell RNA sequencing. LncMN3 expression levels are represented with violet-yellow color scale: higher intensity (yellow) corresponds to higher expression levels, while lower intensity (violet) indicates lower expression levels. Right panel: Dotplot depicting lncMN3 (SERTM2) expression levels in human spinal cord cell populations. Y-axis depicts identified cell populations while x-axis depicts for each cell population the fraction of cells expressing the gene. LncMN3 expression levels are represented with yellow-red color scale: higher intensity (dark red) corresponds to higher expression levels, while lower intensity (yellow) indicates lower expression levels. The expression of lncMN3 in MNs is highlighted by the orange box.

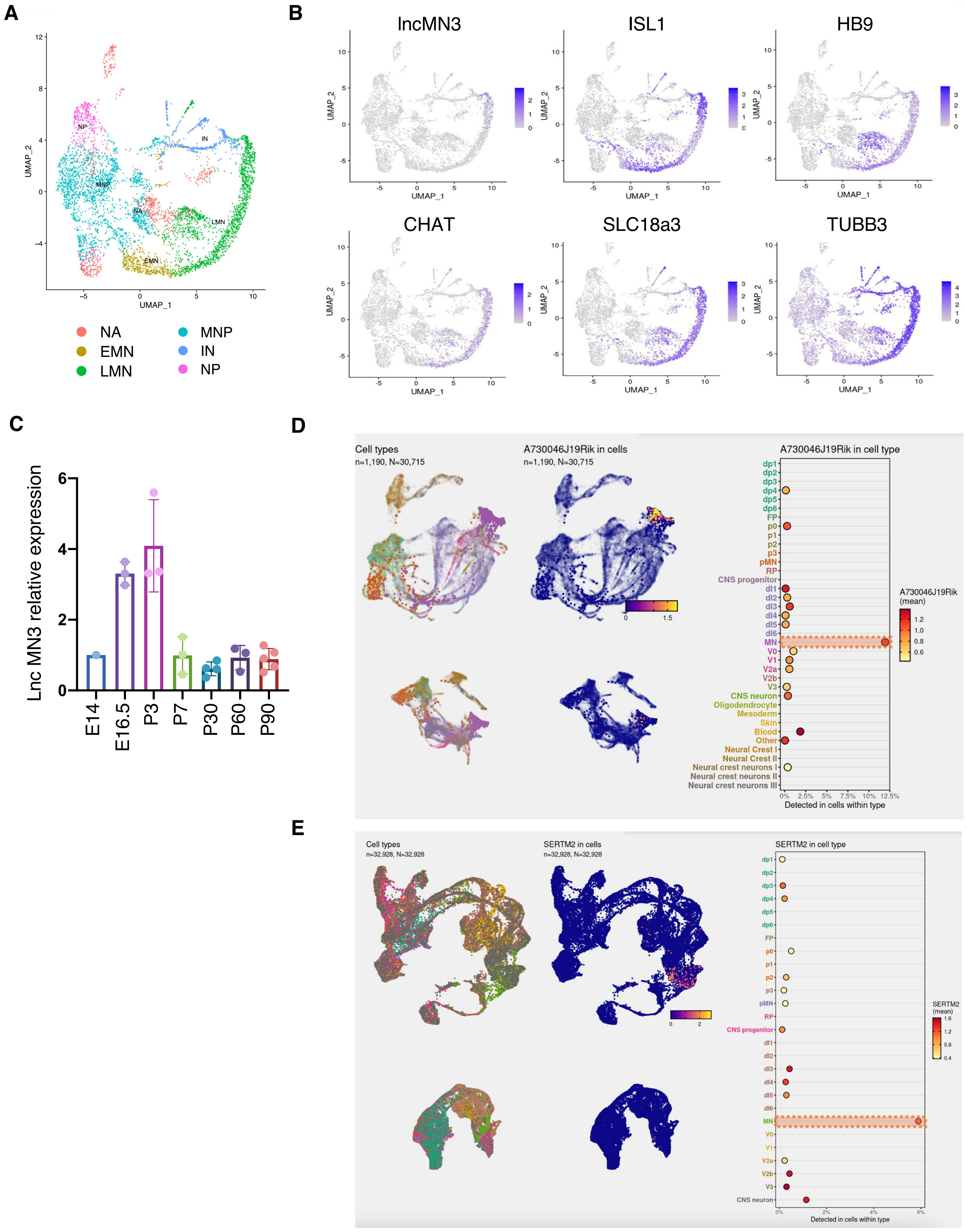
A
B
C
D
E
NA
EMN
LMN
MNP
IN
NP
lncMN3
ISL1
HB9
CHAT
SLC18a3
TUBB3
UMAP_1
UMAP_2
Lnc MN3 relative expression
E14
E16.5
P3
P7
P30
P60
P90
Cell types
n=1,190, N=30,715
A730046J19Rik in cells
A730046J19Rik in cell type
A730046J19Rik (mean)
Detected in cells within type
Cell types
n=32,928, N=32,928
SERTM2 in cells
SERTM2 in cell type
SERTM2 (mean)

 © The Author(s)

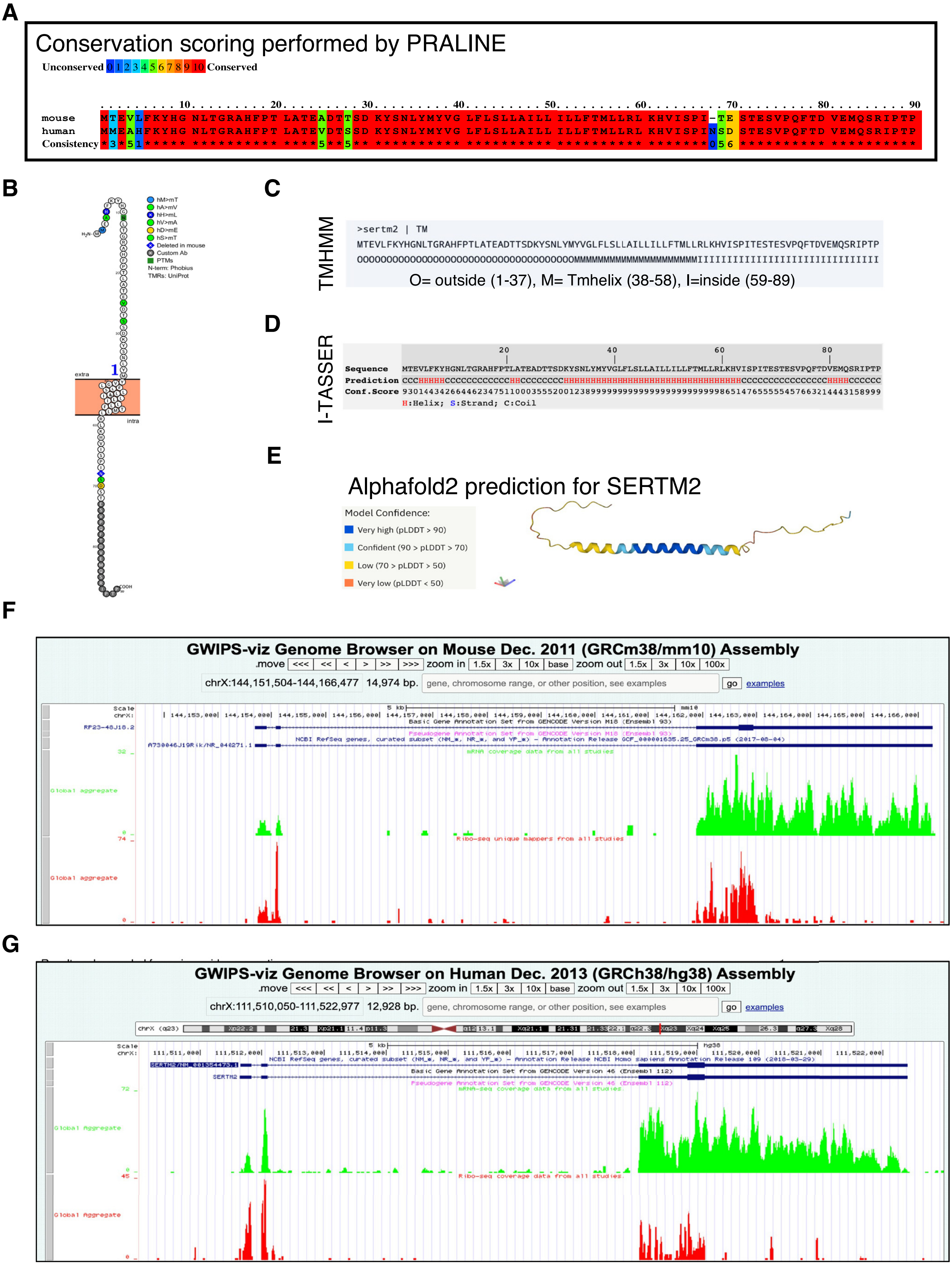
A
Conservation scoring performed by PRALINE
Unconserved 0 1 2 3 4 5 6 7 8 9 10 Conserved
mouse MTEVLFKYHG NLTGRAHFPT LATEADTTSD KYSNLYMYVG LFLSLLAILL ILLFTMLLRL KHVISPI-TE STESVPQFTD VEMQSRIPTP
human MMEAHFKYHG NLTGRAHFPT LATEVDTSSD KYSNLYMYVG LFLSLLAILL ILLFTMLLRL KHVISPINSD STESVPQFTD VEMQSRIPTP
Consistency
B
hM>mT
hA>mV
hH>mL
hV>mA
hD>mE
hS>mT
Deleted in mouse
Custom Ab
PTMs
N-term: Phobius
TMRs: UniProt
extra
intra
COOH
C
TMHMM
>sertm2 | TM
MTEVLFKYHGNLTGRAHFPTLATEADTTSDKYSNLYMYVGLFLSLLAILLILLFTMLLRLKHVISPITESTESVPQFTDVEMQSRIPTP
O= outside (1-37), M= Tmhelix (38-58), I=inside (59-89)
D
I-TASSER
Sequence MTEVLFKYHGNLTGRAHFPTLATEADTTSDKYSNLYMYVGLFLSLLAILLILLFTMLLRLKHVISPITESTESVPQFTDVEMQSRIPTP
Prediction
Conf.Score
H:Helix; S:Strand; C:Coil
E
Alphafold2 prediction for SERTM2
Model Confidence:
Very high (pLDDT > 90)
Confident (90 > pLDDT > 70)
Low (70 > pLDDT > 50)
Very low (pLDDT < 50)
F
GWIPS-viz Genome Browser on Mouse Dec. 2011 (GRCm38/mm10) Assembly
chrX:144,151,504-144,166,477 14,974 bp.
gene, chromosome range, or other position, see examples
go examples
Basic Gene Annotation Set from GENCODE Version M18 (Ensembl 93)
NCBI RefSeq genes, curated subset (NM_*, NR_*, and YP_*) - Annotation Release GCF_000001635.25_GRCm38.p5 (2017-08-04)
mRNA coverage data from all studies
Global aggregate
Ribo-seq unique mappers from all studies
Global aggregate
G
GWIPS-viz Genome Browser on Human Dec. 2013 (GRCh38/hg38) Assembly
chrX:111,510,050-111,522,977 12,928 bp.
gene, chromosome range, or other position, see examples
go examples
SERTM2
Basic Gene Annotation Set from GENCODE Version 46 (Ensembl 112)
mRNA-seq coverage data from all studies
Global Aggregate
Ribo-seq coverage data from all studies
Global Aggregate

Figure EV2. LncMN3 Coding potential.

(**A**) Alignment of mouse and human predicted lncMN3-derived micropeptides performed using PRALINE algorithm (Praline scores range: 0–10). (**B–E**) A conserved transmembrane (TM) domain is predicted in both species by different algorithms (PROTTER—**B**; TMHMM—**C**; TASSER—**D**; and Alphafold2—**E**). (**B**) The SERTM2 sequence recognized by the custom antibody is depicted in gray. Amino acid substitutions between human and mouse sequences are also indicated with the same color code used in (**A**). (**F**) Screenshot of ribosome profiling analysis using the GWIPS-viz browser for mouse A730046j19Rik (lncMN3) locus. Red histogram represents ribo-seq coverage data from all studies (Elongating Ribosomes-Footprints). Green histogram represents mRNA-seq coverage data from all studies (mRNA-seq Reads). (**G**) Screenshot of ribosome profiling analysis using the GWIPS-viz browser for human SERTM2 locus. Red histogram represents ribo-seq coverage data from all studies (Elongating Ribosomes-Footprints). Green histogram represents mRNA-seq coverage data from all studies (mRNA-seq Reads).

 © The Author(s)

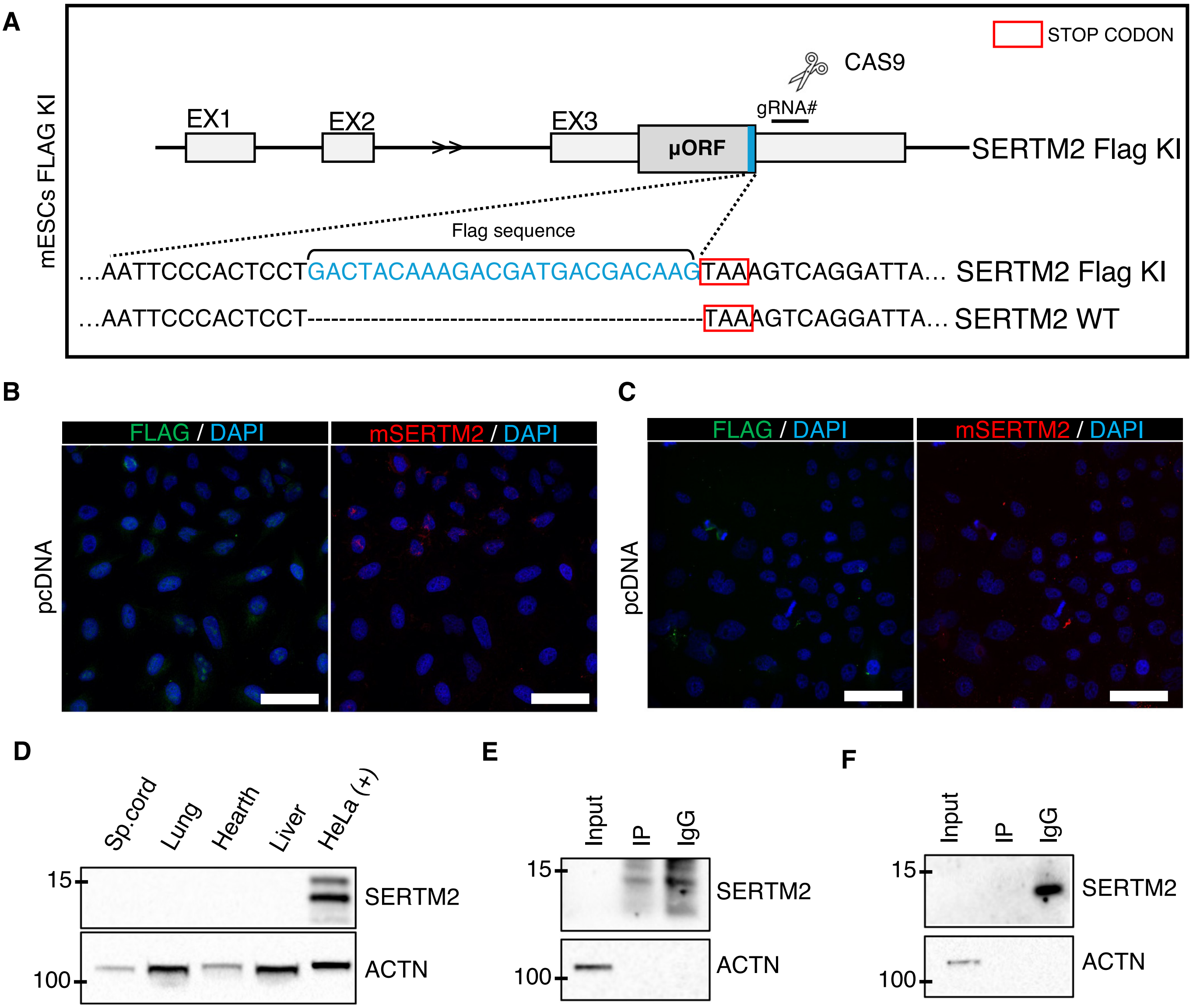

Figure EV3. SERTM2 is a type I transmembrane micropeptide.

(**A**) Schematic representation of CRISPR/CAS9 genome editing strategy used to insert a C-terminal FLAG-tag in frame with the sORF in mESCs. (**B**) Immunofluorescence for FLAG (green signal, left panel) and SERTM2 (red signal, right panel) detection performed on HeLa cells transfected with an empty plasmid (pcDNA). Before immunofluorescence, samples were permeabilized with (0.1%) Triton X-100 detergent. Nuclei were stained with DAPI (blue signal). Scale bar corresponding to 50 μm. Representative experiment of 3 biological replicates. (**C**) Immunofluorescence for FLAG (green signal, left panel) and SERTM2 (red signal, right panel) detection performed on HeLa cells transfected with an empty plasmid (pcDNA). Triton X-100 permeabilization treatment was omitted. Scale bar corresponding to 50 μm. Representative experiment of 3 biological replicates. (**D**) Western blot analyses using antibodies against SERTM2 performed on protein extracts obtained from the indicated tissues. A protein extract obtained from HeLa overexpressing SERTM2-FLAG was used as positive control. ACTN was used as loading control. Representative experiment of 3 biological replicates. (**E**) Representative western blot with anti-SERTM2 antibodies on proteins obtained after SERTM2 immunoprecipitation from 3-month-old mouse liver extract (P90). Input sample accounts for 1.5% of the extract. ACTN was used as negative control. Representative experiment of 2 biological replicates. (**F**) Representative western blot with anti-SERTM2 antibodies on proteins obtained after SERTM2 immunoprecipitation from 3 months old mouse lung extract (P90). Input sample accounts for 1.5% of the extract. ACTN was used as negative control. Representative experiment of 3 biological replicates.

© The Author(s)

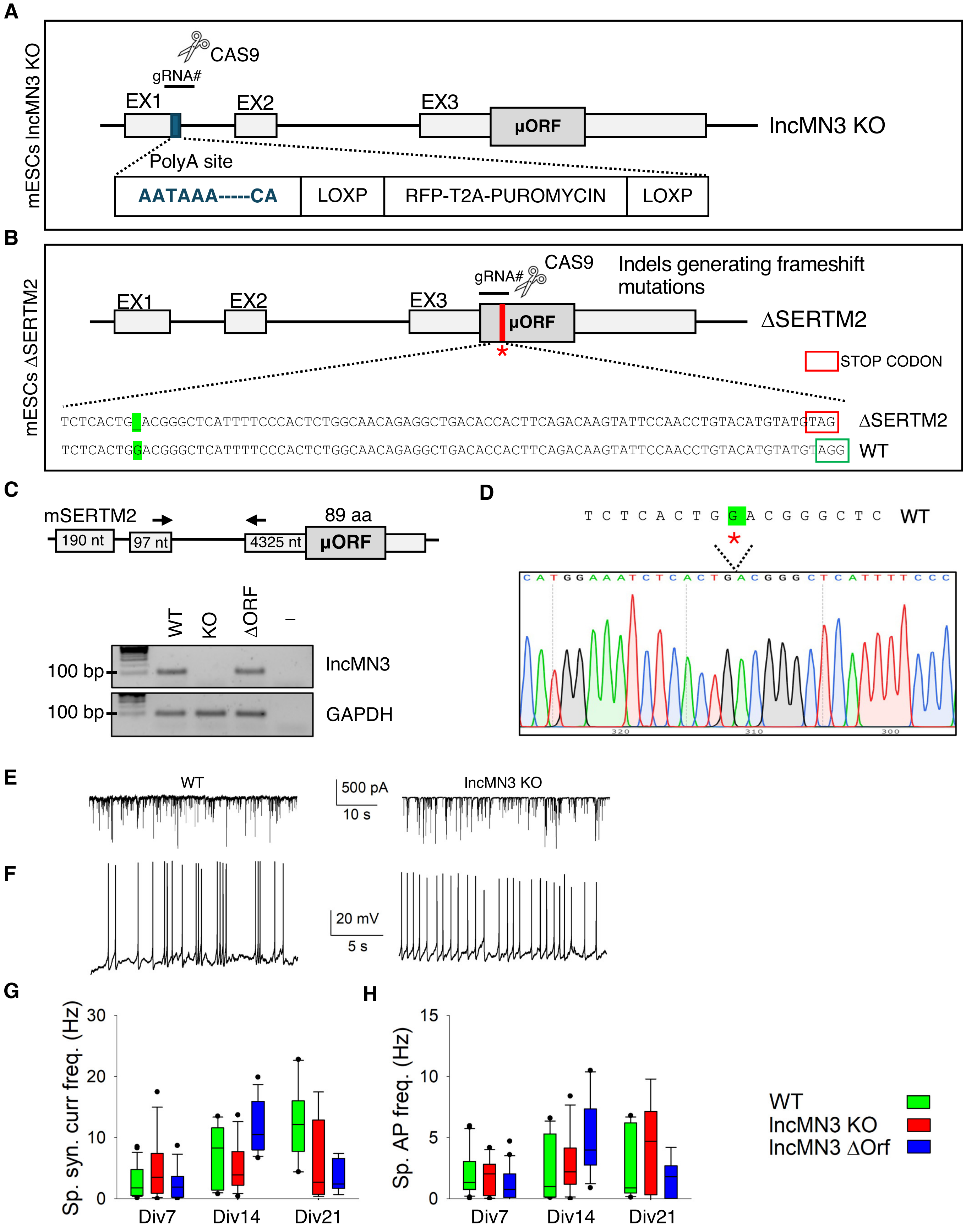
A
mESCs lncMN3 KO
CAS9
gRNA#
EX1
EX2
EX3
µORF
lncMN3 KO
PolyA site
AATAAA-----CA
LOXP
RFP-T2A-PUROMYCIN
LOXP
B
mESCs ΔSERTM2
CAS9
gRNA#
Indels generating frameshift mutations
EX1
EX2
EX3
µORF
ΔSERTM2
STOP CODON
TCTCACTG_ACGGGCTCATTTTCCCACTCTGGCAACAGAGGCTGACACCACTTCAGACAAGTATTCCAACCTGTACATGTATGTAG ΔSERTM2
TCTCACTGGACGGGCTCATTTTCCCACTCTGGCAACAGAGGCTGACACCACTTCAGACAAGTATTCCAACCTGTACATGTATGTAGG WT
C
mSERTM2
89 aa
190 nt
97 nt
4325 nt
µORF
WT
KO
ΔORF
–
100 bp
lncMN3
100 bp
GAPDH
D
T C T C A C T G G A C G G G C T C WT
C A T G G A A A T C T C A C T G A C G G G C T C A T T T T C C C
320
310
300
E
WT
500 pA
10 s
lncMN3 KO
F
20 mV
5 s
G
Sp. syn. curr freq. (Hz)
30
20
10
0
Div7
Div14
Div21
H
Sp. AP freq. (Hz)
15
10
5
0
Div7
Div14
Div21
WT
lncMN3 KO
lncMN3 ΔOrf

 © The Author(s)

Figure EV4. Block of SERTM2 expression does not alter the spontaneous activity of mESC-derived neurons.

(**A**) Schematic representation of CRISPR/CAS9 genome editing strategy used to obtain lncMN3-KO mESC. (**B**) Schematic representation of CRISPR/CAS9 genome editing strategy used to obtain ΔSERTM2 mESCs. (**C**) Upper panel: schematic representation of A730046J19Rik locus. Lower panel: sqRT-PCR showing the expression of lncMN3 transcript on RNA from WT, KO and ΔORF clones. Used oligonucleotide are represented by arrows in the upper panel. GAPDH was used as control. Representative experiment of 3 biological replicates. (**D**) Sequencing chromatogram of the edited sORF region showing the indel generating the frameshift mutation. WT sequence is indicated above the chromatogram. (**E**) Typical traces of spontaneous synaptic currents recorded in voltage-clamp configuration at -70 mV from a WT (left) and a lncMN3-KO (right) neuron. (**F**) Typical traces of spontaneous activity of membrane potential recorded in current-clamp configuration from a WT (left) and a lncMN3-KO (right) neuron. (**G**) Box and whisker plots representing the frequency of spontaneous synaptic currents of WT, lncMN3-KO and lncMN3-ΔORF neurons, at DIV 7 ($n = 24$, $n = 13$, $n = 20$ for WT, lncMN3-KO and lncMN3-ΔORF, respectively), 14 ($n = 15$, $n = 18$, $n = 13$ for WT, lncMN3-KO and lncMN3-ΔORF, respectively) and 21 ($n = 11$, $n = 8$, $n = 7$ for WT, lncMN3-KO and lncMN3-ΔORF, respectively), as indicated. The box plots display the 90th and 10th percentiles at the whiskers, the 75th and 25th percentiles at the boxes, and the median at the central line. Black circles represent outlier data outside the 10th and 90th percentiles. Statistical analyses were performed using one-way ANOVA. Same cells as Fig. 4. Please note no significant difference. (**H**) Box and whisker plots representing the frequency of spontaneous APs of WT, lncMN3-KO and lncMN3-ΔORF neurons, at DIV 7 ($n = 24$, $n = 13$, $n = 20$ for WT, lncMN3-KO and lncMN3-ΔORF, respectively), 14 ($n = 15$, $n = 18$, $n = 13$ for WT, lncMN3-KO and lncMN3-ΔORF, respectively) and 21 ($n = 11$, $n = 8$, $n = 7$ for WT, lncMN3-KO and lncMN3-ΔORF, respectively), as indicated. The box plots display the 90th and 10th percentiles at the whiskers, the 75th and 25th percentiles at the boxes, and the median at the central line. Black circles represent outlier data outside the 10th and 90th percentiles. Statistical analyses were performed using one-way ANOVA. Same cells as Fig. 4. Please note no significant difference.

© The Author(s)

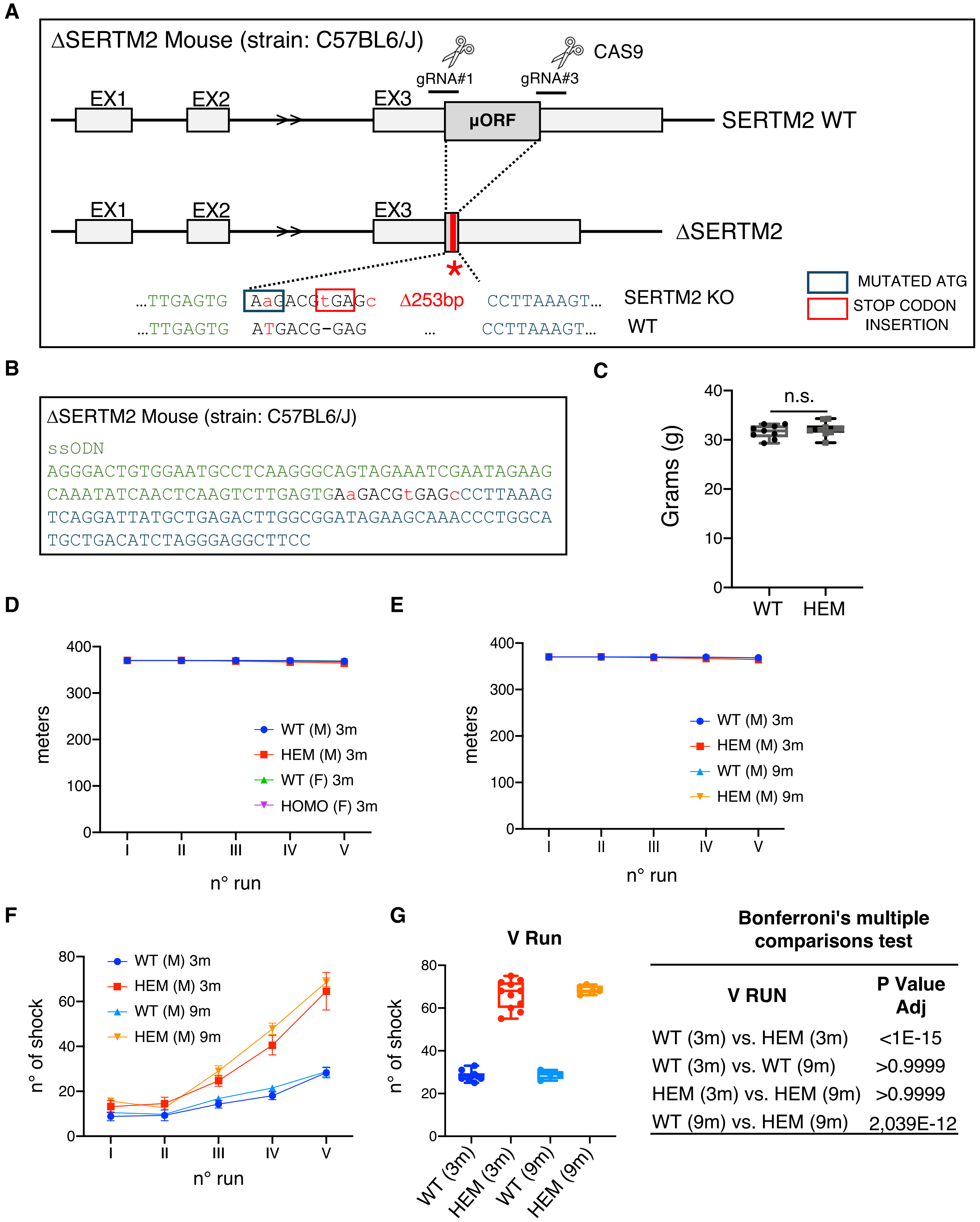
A
ΔSERTM2 Mouse (strain: C57BL6/J)
CAS9
gRNA#1
gRNA#3
EX1
EX2
EX3
μORF
SERTM2 WT
ΔSERTM2
...TTGAGTG AaGACGtGAGc Δ253bp CCTTAAAGT... SERTM2 KO
...TTGAGTG ATGACG-GAG ... CCTTAAAGT... WT
MUTATED ATG
STOP CODON INSERTION
B
ΔSERTM2 Mouse (strain: C57BL6/J)
ssODN
AGGGACTGTGGAATGCCTCAAGGGCAGTAGAAATCGAATAGAAG
CAAATATCAACTCAAGTCTTGAGTGAaGACGtGAGcCCTTAAAG
TCAGGATTATGCTGAGACTTGGCGGATAGAAGCAAACCCTGGCA
TGCTGACATCTAGGGAGGCTTCC
C
Grams (g)
n.s.
WT
HEM
D
meters
n° run
WT (M) 3m
HEM (M) 3m
WT (F) 3m
HOMO (F) 3m
E
meters
n° run
WT (M) 3m
HEM (M) 3m
WT (M) 9m
HEM (M) 9m
F
n° of shock
n° run
WT (M) 3m
HEM (M) 3m
WT (M) 9m
HEM (M) 9m
G
V Run
n° of shock
WT (3m)
HEM (3m)
WT (9m)
HEM (9m)
Bonferroni's multiple comparisons test
V RUN
P Value Adj
WT (3m) vs. HEM (3m) <1E-15
WT (3m) vs. WT (9m) >0.9999
HEM (3m) vs. HEM (9m) >0.9999
WT (9m) vs. HEM (9m) 2,039E-12

© The Author(s)

Figure EV5. SERTM2-KO mouse model characterization.

(**A**) Schematic representation of CRISPR/CAS9 genome editing strategy used to obtain ΔSERTM2 mice. (**B**) ssODN donor sequence used to obtain ΔSERTM2 mice in the CRISPR/CAS9 genome editing strategy. (**C**) Mean body weight for 3-month-old male WT ($n = 9$) and HEM ($n = 10$) mice. Data are represented as a scatter plot. Each point corresponds to a single value. Statistical analyses were performed using Student *t* test. (**D**) Treadmill test performance represented by meters per run on the following 4 groups of 3 months old mice: WT male ($n = 10$), HEM males ($n = 12$), WT female ($n = 9$), HOMO female ($n = 9$). Treadmill test was repeated twice a week for a total of five runs (I–V) and run meters were recorded. Values are mean ± SD. (**E**) Treadmill test performance represented by meters per run on the following 4 groups of male mice: WT 3 m ($n = 10$), HEM 3 m ($n = 12$), WT 9 m ($n = 4$), HEM 9 m ($n = 5$). Treadmill test was repeated twice a week for a total of five runs (I–V) and run meters were recorded. Values are mean ± SD. (**F**) Treadmill test performance represented by shock numbers assessed on the following 4 groups of male mice: WT 3 m ($n = 10$), HEM 3 m ($n = 12$), WT 9 m ($n = 4$), HEM 9 m ($n = 5$). Treadmill test was repeated twice a week for a total of five runs (I–V) and shock numbers were recorded. Values are mean ± SD. (**G**) Number of shocks received in the fifth run by each group of mice described in (**F**): WT 3 m ($n = 10$), HEM 3 m ($n = 12$), WT 9 m ($n = 4$), HEM 9 m ($n = 5$). The box plots show the minimum and maximum values at the whiskers, the 75th and 25th percentiles at the boxes, and the median at the central line. Statistical analyses were performed using one-way ANOVA followed by Bonferroni's Multiple Comparison test. The adjusted *P* values are indicated in the right panel.

© The Author(s)
